# Strength of a cement-based dental material: Early age testing and first micromechanical modeling at mature age

**DOI:** 10.3389/fbioe.2023.1047470

**Published:** 2023-03-09

**Authors:** Petr Dohnalík, Christian Hellmich, Gilles Richard, Bernhard L. A. Pichler

**Affiliations:** ^1^ Institute for Mechanics of Materials and Structures, TU Wien (Vienna University of Technology), Vienna, Austria; ^2^ Septodont, Saint-Maur-des-Fossés, France

**Keywords:** compressive strength, early-age strength evolution, continuum micromechanics, lognormal stiffness distribution, lognormal strength distribution, degree of utilization, cement-based dental material

## Abstract

The compressive strength evolution of 37 centigrade-cured Biodentine, a cement-based dental material, is quantified experimentally by crushing cylindrical specimens with length-to-diameter ratios amounting to 1.84 and 1.34, respectively, at nine different material ages ranging from 1 h to 28 days. After excluding strength values significantly affected by imperfections, formulae developed for concrete are i) adapted for inter- and extrapolation of measured strength values, and ii) used for quantification of the influence of the slenderness of the specimens on the compressive strength. The microscopic origin of the macroscopic uniaxial compressive strength of *mature* Biodentine is investigated by means of a micromechanics model accounting for lognormal stiffness and strength distributions of two types of calcite-reinforced hydrates. The following results are obtained: The material behavior of Biodentine is non-linear in the first few hours after production. After that, Biodentine behaves virtually linear elastic all the way up to sudden brittle failure. The strength evolution of Biodentine can be well described as the exponential of a function involving the square root of the inverse of the material age. The genuine *uniaxial* compressive strength evolution can be quantified using a correction formula taken from a standard for testing of concrete, which accounts for length-to-diameter ratios of cylindrical samples deviating from 2. Multiscale modeling suggests that 63% of the overall material volume, occupied by dense calcite-reinforced hydration products, fail virtually simultaneously. This underlines the highly optimized nature of the studied material.

## 1 Introduction

The compressive strength is a popular maturity and performance indicator for cementitious materials. Rich knowledge regarding strength testing and modeling is available in the construction sector. Herein, this knowledge will be used to increase the understanding of the maturation and the performance of the cement-based dental material Biodentine.

Biodentine is made from a cementitious powder and a mixing liquid. The chemical composition of the powder was quantified by means of X-ray diffraction analyses with Rietveld refinement, see, e.g., (Camilleri et al., 2013; Grazziotin-Soares et al., 2019; Li et al., 2019): Some 74 wt% are tricalcium silicate (Ca_3_SiO_5_), some 4.5 wt% dicalcium silicate (Ca_2_SiO_4_), some 16.5 wt% calcium carbonate (CaCO_3_), and some 5 wt% zirconium dioxide (ZrO_2_). The mixing liquid contains water (H_2_O), calcium chloride (CaCl_2_), and a hydrosoluble polycarboxylate-based polymer (Septodont, 2014).

In the present paper, two main objectives are pursued: i) An experimental campaign will be carried out in order to study the early-age evolution of the compressive strength of Biodentine, with a particular focus on the influence of the slenderness of cylindrical specimens on the obtained strength values. ii) A multiscale model will be developed in order to investigate the stress-to-strength ratio experienced by microscopic hydrate phases of mature Biodentine, provided that the material is subjected to loading which is equal to its uniaxial compressive strength. The underlying motivation will be explained in the following paragraphs.

**FIGURE 1:**
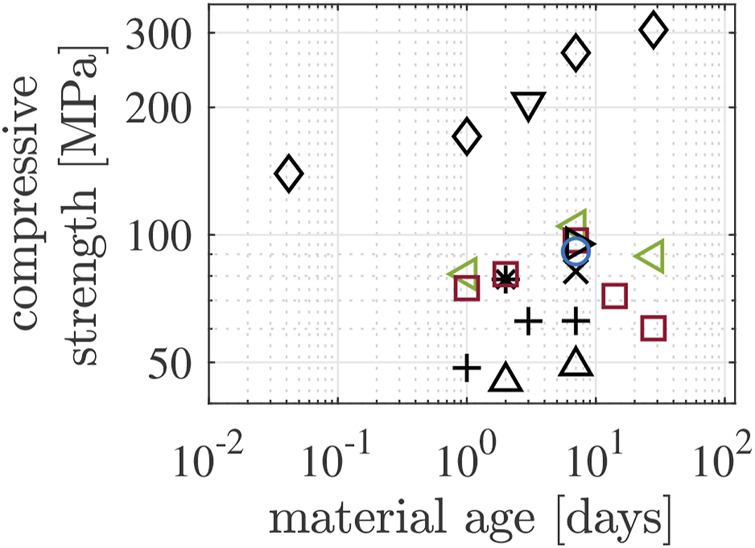
Compressive strength values of Biodentine either cured in distilled water or at/close to 100% relative humidity, see Table 1; different markers correspond to different authors: △ Kayahan et al. (2013), ⊳ Elnaghy (2014), + Jang et al. (2014), *♢* Butt et al. (2014), * Dawood et al. (2015), ⊲ Moreno-Vargas et al. (2017), ▽ Subramanyam and Vasantharajan (2017), ◦ Keskin et al. (2019), □ Atmeh (2020); different colors correspond to different inner mold dimensions: black to length = 6 mm and diameter = 4 mm, red to length = 10 mm and diameter = 5 mm, green to length = 6 mm and diameter = 3 mm, blue to length = 5 mm and diameter = 4 mm.

Compressive strength values of Biodentine show a large scatter, even when focusing on results from studies in which the specimens were either cured under water or exposed to air with relative humidity *RH* ≥ 95%, see, e.g., (Rajasekharan et al., 2014; Rajasekharan et al., 2018; Primus et al., 2019) and references therein. Strength values obtained 1 day and 7 days after production range from 48.5 MPa to 170 MPa and from 49.1 MPa to 269 MPa, respectively, see Figure 1 and Table 1. The experimental part of the present study is designed to address the following two reasons for this scatter.

• Imperfections reduce the ultimate load sustained by specimens relative to the compressive strength of the material they are made from, e.g., air bubbles entrapped during casting, problems regarding the co-planarity of the loaded surfaces of test specimens, etc.
• The length-to-diameter ratio of cylindrical samples influences the results of compressive strength testing, because shear stresses resulting from friction in the interfaces between the load platens and the specimens increase the ultimate loads sustained by the specimens relative to the compressive strength of the material they are made from (Amieur, 1994). Notably, the strength values illustrated in Figure 1 refer to specimens with length-to-diameter ratios ranging from 1.25 to 2.00.

**TABLE 1:** Overview of details regarding the compressive strength data illustrated in Figure 1.

Publication (year)	Curing cond.	Load. rate	Spec. size L/D [mm/mm]	No. spec.	Time inst. [day]
Kayahan et al. (2013)	100% RH	1 mm/min	6/4	20	7
Elnaghy (2014)	in wet gauze	0.5 mm/min	6/4	15	7
Jang et al. (2014)	in dist. water	1 mm/min	6/4	10	1, 3, 7
Butt et al. (2014)	in dist. water	0.5 mm/min	6/4	10	1/24, 1, 7, 28
Natale et al. (2015)	100% RH	1 mm/min	6/4	10	2, 7
Dawood et al. (2015)	95% RH	1 mm/min	6/4	10	2
Moreno-Vargas et al. (2017)	95% RH	1 mm/min	6/3	10	1, 7, 28
Subramanyam and Vasantharajan (2017)	100% RH	1 mm/s	6/4	5	3
Keskin et al. (2019)	95% RH	1 mm/min	5/4	15	7
Atmeh (2020)	100% RH	50 N/min	10/5	12	1, 2, 7, 14, 28

Herein, the early-age strength evolution of Biodentine will be characterized using cylindrical samples with *two different length-to-diameter ratios*. Every single test will be checked for indicators suggesting the influence of imperfections. Tests significantly affected by imperfections will be excluded. The remaining relations between strength and material age will allow for studying the influence of the slenderness of the specimens on the early-age strength evolution.

The uniaxial compressive strength of well-hardened construction “cement paste” reaches values ranging up to some 100 MPa (Pichler et al., 2013a)^1^. The strength of Biodentine outperforms these values by a factor of virtually three. This provides the motivation for the multiscale modeling part of the present study. It is aimed at investigating the microscopic origin of the macroscopic uniaxial compressive strength of mature Biodentine. Strength modeling builds on the results of two preceding studies, as explained next. The microstructure of mature Biodentine consists of zirconium dioxide, residual calcium silicates, and two populations of calcite-reinforced hydration products (Dohnalík et al., 2021). The expression “populations” is to be understood statistically, because more than 5,000 nanoindentation tests into hardened Biodentine underlined that the stiffness and strength of both types of hydrates are lognormally distributed (Dohnalík et al., 2021). The stiffness distributions were accounted for in the first continuum mechanics model for the elastic stiffness of well-hardened Biodentine (Dohnalík et al., 2022). Weak microstructural grain boundaries were modeled by means of isotropically oriented, circular slit cracks. The microcrack density was identified such that the model reproduces the isotropic elastic stiffness of well-hardened Biodentine, as characterized by means of ultrasonic pulse velocity measurements using both longitudinal and transversal waves with frequencies in the interval from 50 kHz to 20 MHz (Dohnalík et al., 2022). Herein, the described model will be used for quantifying microstresses experienced by the hydration products, as a function of their stiffness, provided that a macroscopic sample of Biodentine is subjected to loading which is equal to its uniaxial compressive strength. The computed microstress distribution will be eventually compared with a lognormal microstrength distribution which is correlated with the nanoindentation-derived distributions of indentation modulus and hardness.

The present paper is structured as follows: Section 2 is focused on early-age strength testing of Biodentine and the analysis of the results. In Section 3, the early-age strength data of Biodentine is evaluated exploiting knowledge from compressive strength testing of concrete. Section 4 refers to multiscale modeling of the uniaxial compressive strength of mature Biodentine. Section 5 provides a discussion of the results. Section 6 closes the paper with conclusion.

## 2 Compressive strength testing from early to mature material ages

In the following, i.e. from here throughout Section 4.2, compressive forces and stresses as well as the related shortening of the specimens will be quantified by positive numbers.

### 2.1 Production and storage of cylindrical specimens

Specimens of Biodentine were produced according to the recommendations of the manufacturer. Capsules containing the dry powder were opened. 173 μL of the mixing liquid were dripped onto the powder. For preciseness and repeatability reasons, a micropipette (Handy Step, Mettler Toledo, United States) was used, rather than the clinically used dropper provided by the manufacturer, because variations in the solid-to-liquid mass ratio lead to considerable changes of the material properties (Pires et al., 2021). The capsules were closed, shaken in an automatic mixer (SYG-200, Hangzhou Sifang Medical Apparatus Co., Ltd.) at 4,600 rpm for 30 s to ensure a uniform distribution of the constituents of Biodentine throughout the microstructures of the produced specimens, and re-opened. The freshly produced cement paste was cast into cylindrical polytetrafluoroethylene molds with two different length-to-diameter ratios. The slenderer molds had a length = 10 mm and a diameter = 5 mm. The less slender molds had a length = 6 mm and a diameter = 4 mm. The corresponding specimens are referred to as “5/10 specimens” and “4/6 specimens,” respectively.

**FIGURE 2:**
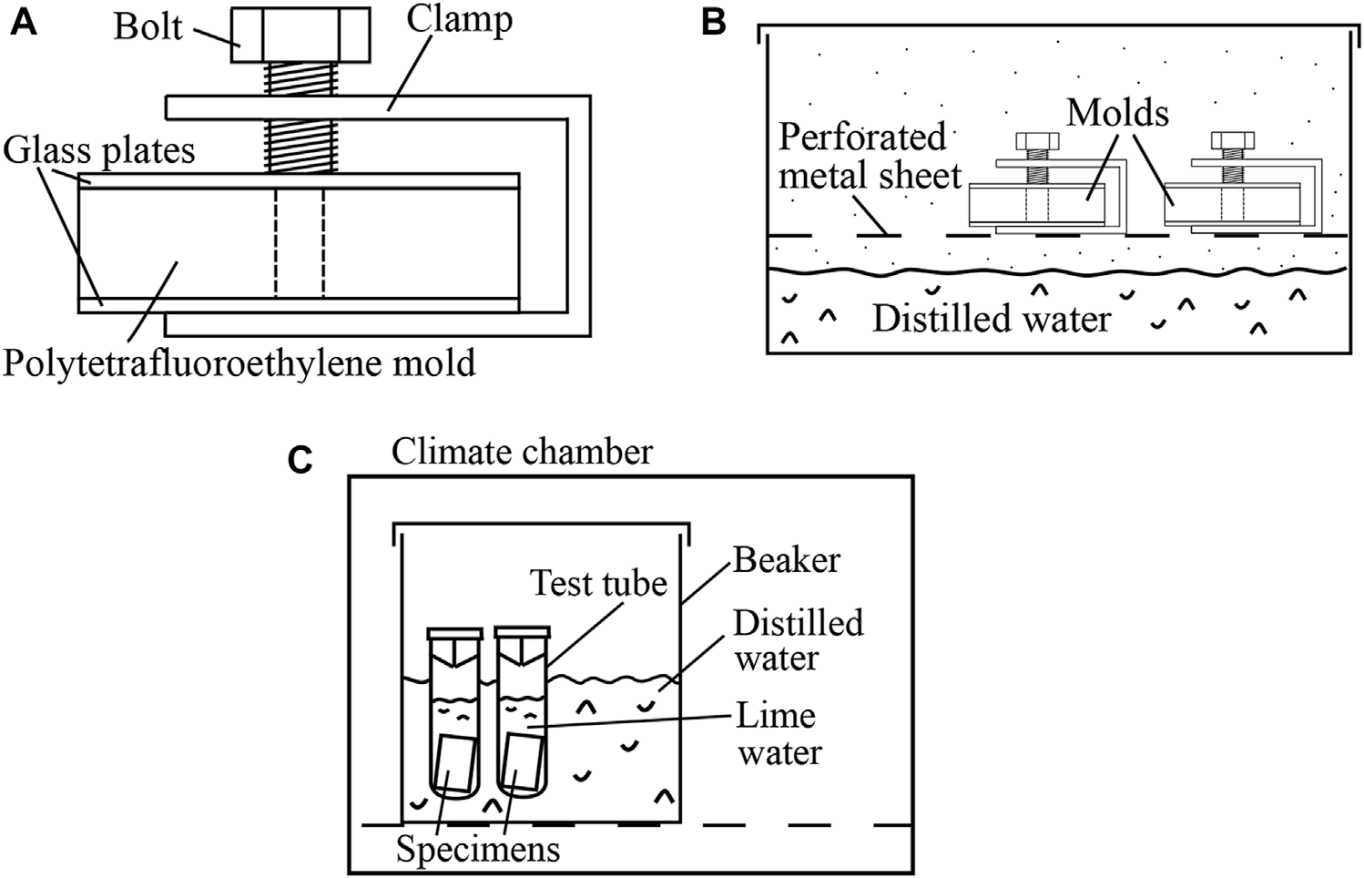
**(A, B)** Storage of specimens of Biodentine during their transition from a moldable gel into a solid: **(A)** specimen inside a polytetrafluoroethylene mold, covered by two glass plates which were secured by means of a U-shaped clamp and a screw, **(B)** the clamped molds were stored above a water bath tempered to 37°C. **(C)** Storage of solid specimens until they were scheduled for destructive compressive strength testing: the specimens were stored in lime-saturated solution inside a temperature-controlled environment.

In order to allow the material to transform from a moldable gel into a solid, the molds were covered by glass plates (= microscope slides). The latter were secured by means of a U-shaped clamp and a screw, see Figure 2A. This assembly was stored inside a container, above a water bath tempered to 37°C, see Figure 2B. After 20 min, the solidified specimens were taken out of their molds.

In order to protect the specimens against drying and calcium leaching, they were inserted into test tubes filled with lime-saturated solution tempered to 37°C. The test tubes were closed and put into a beaker containing distilled water. The beaker was stored in a climate chamber (MOV-112F, Sanyo, Japan), see Figure 2C. Both the distilled water and the climate chamber were tempered to 37°C, see Figure 2C. In this configuration, the specimens hardened until they were scheduled for testing.

In order to prepare the specimens for destructive compressive testing, they were taken out of the lime-saturated solution and processed as follows. In order to come up with coplanarity of the two opposite circular surfaces, they were ground by means of a polishing machine (LaboPol-5, Struers, Germany) operated at 300 revolutions per minute, using silicon carbide grinding paper with a grain size of 15 μm (Struers Grit 1,200). During grinding, the specimens were kept in a holder which was geometrically identical to the molds described above. The length *L* and the diameter *D* of the ground specimens were measured using a digital sliding caliper (CD-S15CK, Mitutoyo, Japan). The 5/10 specimens had an average length = 9.22 mm, see Supplementary Table S1. The 4/6 specimens had an average length = 5.26 mm, see Supplementary Table S2. Thus, the average length-to-diameter ratios of the two types of specimens amounted to

5/10 specimens:⇒L/D=1.84, (1)

4/6 specimens:⇒L/D=1.34. (2)

After polishing, two photographs per specimen were taken, documenting each specimen's two polished surfaces. Finally, the specimens were placed, one after the other, onto the bottom load plate of a universal mechanical testing machine (Criterion C43.104Y, MTS Systems Corporation, United States of America). The cross-head was very slowly moved downwards, under visual control, until the upper load plate was very close to (but not yet in contact with) the specimen.

### 2.2 Destructive compression testing

The mechanical testing machine was employed for performing destructive uniaxial compression experiments under displacement control, realized through software TW Elite v4.6.0.23, MTS Systems Corporation, United States. When it came to the definition of the speed of the cross-head, it was accounted for the fact that the strength of cementitious materials increases with increasing speed of loading, see, e.g., Figure 2 in (Fischer et al., 2014) for strength values of 2-day-old construction “cement paste”. Therefore, *the same speed of loading* had to be prescribed in the present study, in order to achieve direct comparability of strength values obtained with specimens of *different* sizes. To this end, the nominal strain rate was set equal to $\approx 1.5\times 10^{-3}/s$ . This is a typical value for quasi-static testing. As for the execution of the tests, the speed of the cross-head had to be prescribed. This was obtained by multiplying the desired nominal strain rate with the average length of the tested cylinders. Thus, the speed of the cross-head was set equal to 0.83 mm/min for the 5/10 specimens (average length = 9.22 mm), and to 0.50 mm/min for the 4/6 specimens (average length = 5.26 mm). As output, the software of the testing machine delivered histories of the “machine displacement” Δ*L*(*t*), and of the force *F*(*t*) imposed on the tested specimen.

A total of 122 destructive compression tests were performed at material ages ranging from 1 h to 28 days. The resulting ultimate force readings are listed in Tables 2, 3. Therein, the material age is counted from the time instant when the mixing liquid got in contact with the dry powder.

**TABLE 2:** Values of the ultimate compressive forces sustained by the 5/10 specimens, tested at specific material ages.

Material age	Ultimate force *F*_*ult*_ [N] of the specimen *n*						
	1	2	3	4	5	6	7
1 h	1,060	990	942	1,095	810†	849	−
1.5 h	1,940	1,369	1853	1,896	1,630	1,998	1,826
3.0 h	3,081	2,979	2,422⋆	2,673†	2,821	2,790†	−
7.5 h	3,042	2,951⋆	3,201	2,760⋆	2,835⋆	3,259	3,262†
25.5 h	3,006†	2,792⋆	3,498⋆	3,951	4,154	3,899	3,807†
7 days	4,616	5,358	4,024⋆	4,504⋆	4,805⋆	4,518	4,134⋆
7 days‡	5,036	4,815⋆	3,160⋆	3,570⋆	4,082⋆	4,322⋆	3,900⋆
14 days	4,787⋆	4,878⋆	4,446⋆	3,523⋆	4,832⋆	5,371	5,387
14 days‡	4,967⋆	4,786⋆	5,371⋆	5,150	4,127⋆	4,035†	5,577⋆
21 days	5,312†	5,157	4,841	4,936	4,333⋆	4,208⋆	4,570⋆
28 days	4,637⋆	6,220⋆	5,355⋆	5,082⋆	4,538⋆	5,026⋆	2,815⋆

⋆ Experiment discarded because of pre-peak cracking event(s).

† Experiment discarded because of visible microstructural imperfection(s).

‡ The numbering of the specimens continues as *n* + 7, with n given in the second line of the table.

**TABLE 3:** Values of the compressive ultimate forces sustained by the 4/6 specimens, tested at specific material ages.

Material age	Ultimate force *F*_*ult*_ [N] of the specimen *n*						
	1	2	3	4	5	6	7
1.5 h	1,466	1,396	779⋆	1,386	1,402	1,130	1,504
7.5 h	−‡	2,116⋆	2,307	2,734	1,550⋆	1839⋆	2,375
25.5 h	2,290⋆	1951⋆	2,779	2,532	1,648⋆	2,696	2,630†
7 days	2,940⋆	2,701⋆	2,143⋆	2,503⋆	3,267	2,898⋆	2,467⋆
14 days	3,178⋆	3,057⋆	3,714	2,798⋆	3,427	2,960⋆	3,059
21 days	1,352⋆	3,555	3,590⋆	1,662⋆	2,820⋆	−‡	3,244
28 days	2,247⋆	3,268⋆	2,902⋆	3,194	3,236⋆	2,288†	1985⋆

⋆ Experiment discarded because of pre-peak cracking event(s).

† Experiment discarded because of visible microstructural imperfection(s).

‡ Specimens failed during handling, prior to testing.

### 2.3 Production of stress-strain diagrams

The method used to translate the output of the testing machine, i.e., Δ*L*(*t*) and *F*(*t*) histories, into stress-strain diagrams is described next. Dividing the “machine displacement” Δ*L*(*t*) by the initial length of the specimens, *L*, delivers the strain measure which was increased linearly with increasing time during the experiments:

$$\varepsilon \left(t\right)=\frac{\Delta L\left(t\right)}{L}.$$ (3)

Notably, Δ*L*(*t*) is proportional to the rotations of the motor powering the testing machine. As for calibration of the “machine displacement,” the cross-head is *freely* moved up and down. During actual testing, however, the rotations of the motor do not only result in a vertical movement of the cross head (and, therefore, in a shortening of the specimen), but also in deformations of all parts of the testing machine, which connect the specimen and the motor. These parts include the load cell, the cross-head, the two spindles (the rotation of which move the cross-head up or down), the vee-belts connecting the spindles with the gearbox of the machine, and the gearbox itself. If the shortening of a specimen is to be captured accurately, measurements must directly refer to the central part of the specimens, see, e.g., (Karte et al., 2015; Irfan-ul-Hassan et al., 2016). This was impossible in the present study, in which millimeter-sized specimens were tested. Therefore, it must be taken into account that Δ*L*(*t*), provided by the testing machine, and *ɛ*(*t*), quantified according to Eq. 3, overestimate the shortening of the specimen and the strain of the specimens, respectively.

The evolution of the axial normal stress *σ*(*t*) of a tested specimen follows from dividing *F*(*t*) by the cross-sectional area = *d* ^2^ *π*/4 of the specimen:

$$\sigma \left(t\right)=\frac{4\;F\left(t\right)}{d^{2}\pi }.$$ (4)

Notably, the force readings are quantitatively reliable, because both the load cell and the specimen are arranged in series, and the same force *F*(*t*) is transmitted through both of them. Thus, also the stresses at failure (= strength values) are quantitatively reliable.

Although *ɛ*(*t*) according to Eq. 3 overestimates the strain of the specimen, stress-strain diagrams still provide valuable qualitative information.

• Linear stress-strain graphs indicate linear material behavior of the specimens.
• An initial positive curvature of stress-strain graphs indicates imperfect (rather than full-face) initial contact between the specimens and the load platens.
• Discontinuities of stress-strain graphs indicate cracking events.
• A negative curvature of stress-strain graphs indicates pre-peak deterioration of the specimens.

### 2.4 Discarding tests influenced by imperfections

Miniaturized compressive strength tests on cylindrical specimens are a challenging task, because such experiments are prone to suffer from imperfections which reduce the ultimate loads sustained by the specimens. This provides the motivation to discard tests which were evidently affected either by geometric imperfections regarding coplanarity or by pores entrapped during casting. The corresponding procedures are described in the following two paragraphs.

**FIGURE 3:**
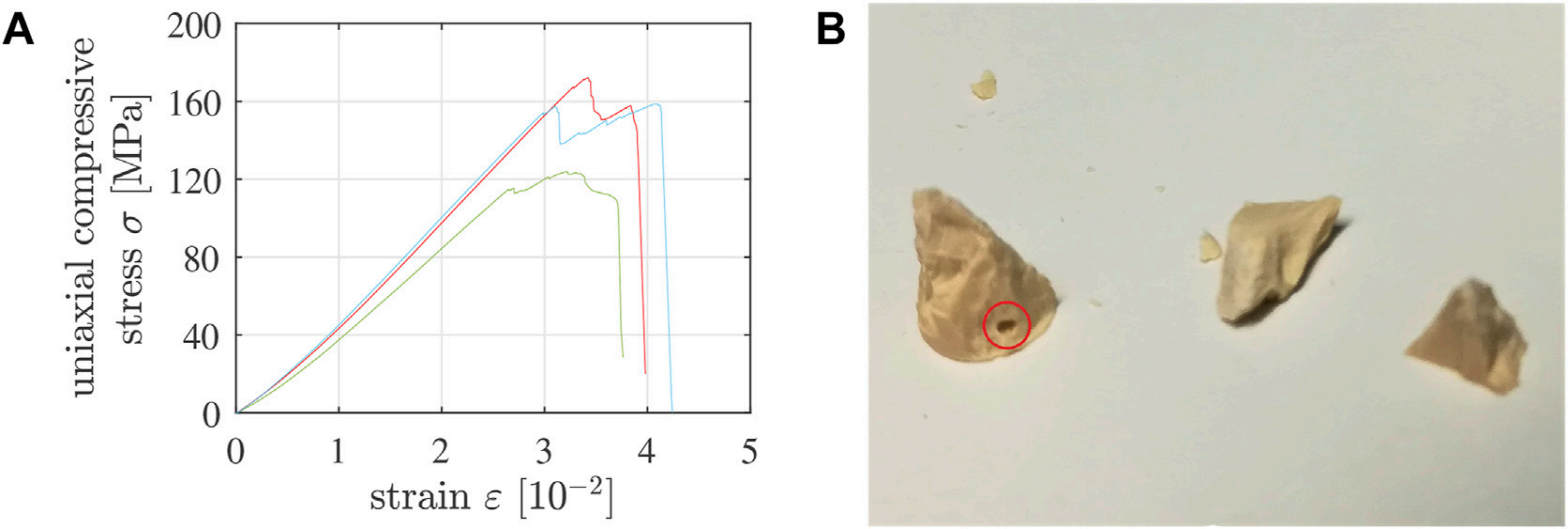
**(A)** Three examples of stress-strain graphs referring to compression tests suffering from pre-peak cracking events: such tests were discarded. **(B)** Fragments of 5/10-specimen No. 7, tested 7.5 h after production, showing a macropore intersected by one of the cracks that had split the specimen: such tests were discarded. Note that “strain” refers to the sum of the absolute values of the compressive axial strain undergone by the specimen and the tensile axial strain undergone by the testing machine.

Geometric imperfections, such as residual roughness or tilt, may lead to partial, rather than full-face, contact between the specimen and the load application system; in particular so during the initial phase of a compression test. This manifests itself in a positive curvature of stress-strain which are small when compared to the finally reached strength, see Figure 3A. Given that geometric imperfections were “small,” progressive increase of compressive loading resulted in the transition to full-face contact and to a virtually linear stress-strain graph. In addition, stress fluctuations (rather than a uniform stress field) occurred inside several of the tested specimens, manifesting themselves by audible cracking events and/or visible spalling, however, in any case by a sudden drop of the sustained force, followed by its re-increase, sometimes even surpassing the level at which the local damage event took place, see Figure 3A. The ultimate force sustained by a specimen which suffered from a pre-peak cracking event is very likely smaller than the ultimate force the specimen would have reached without the damage event. Therefore, we discard all those tests with stress-strain graphs qualitatively similar to the ones shown in Figure 3A. Corresponding values of the ultimate forces are marked with a “⋆”-symbol in Tables 2, 3.

Macropores entrapped unintentionally during casting result in stress concentrations that can trigger premature failure of a specimen. Therefore, the fragments of the specimens were checked for visible defects. In case a macropore was found to be intersected by a macrocrack, see, e.g., Figure 3B, the test was discarded. Corresponding values of the ultimate forces are marked with a “†”-symbol in Tables 2, 3.

### 2.5 Stress-strain diagrams free of significant imperfections

**FIGURE 4:**
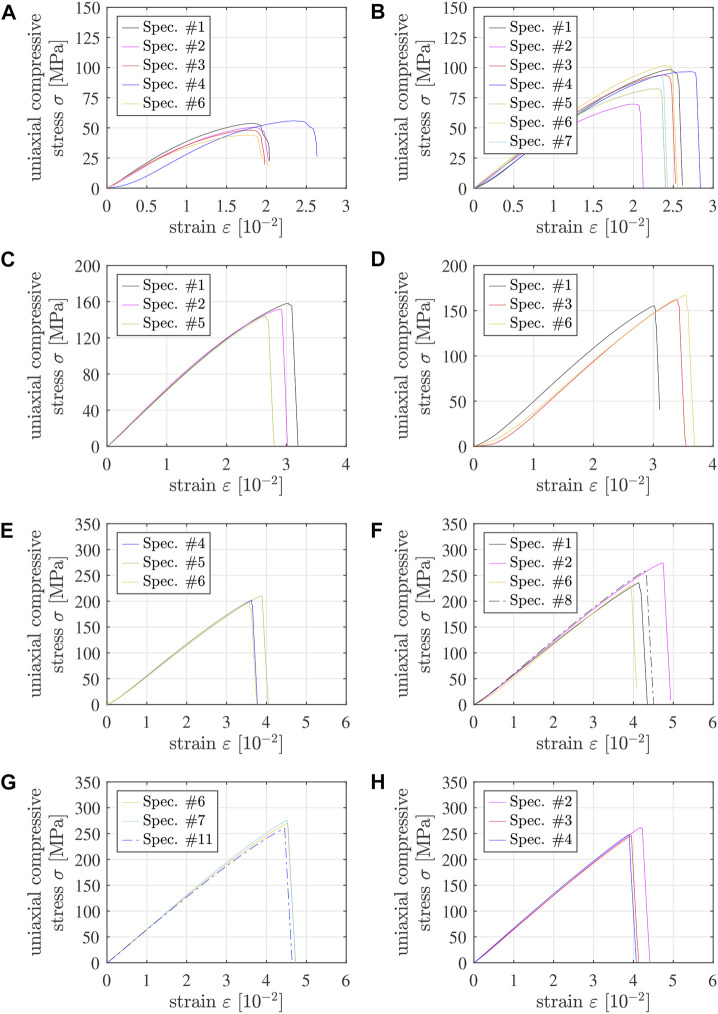
Stress-strain diagrams from destructive compression experiments of 5/10 specimens tested **(A)** 1 h, **(B)** 1.5 h, **(C)** 3 h, **(D)** 7.5 h, **(E)** 25.5 h, **(F)** 7 days, **(G)** 14 days, and **(H)** 21 days after production; the solid graphs refer to specimens #1 throughout #7, with the following color code: #1 - black, #2 - pink, #3 - red, #4 - dark blue, #5 - green, #6 - yellow, #7 - light blue, see also Table 4 and Supplementary Table S1; the dash-dotted lines refer to specimens with numbers greater than 7. Note that “strain” refers to the sum of the absolute values of the compressive axial strain undergone by the specimen and the tensile axial strain undergone by the testing machine.

**FIGURE 5:**
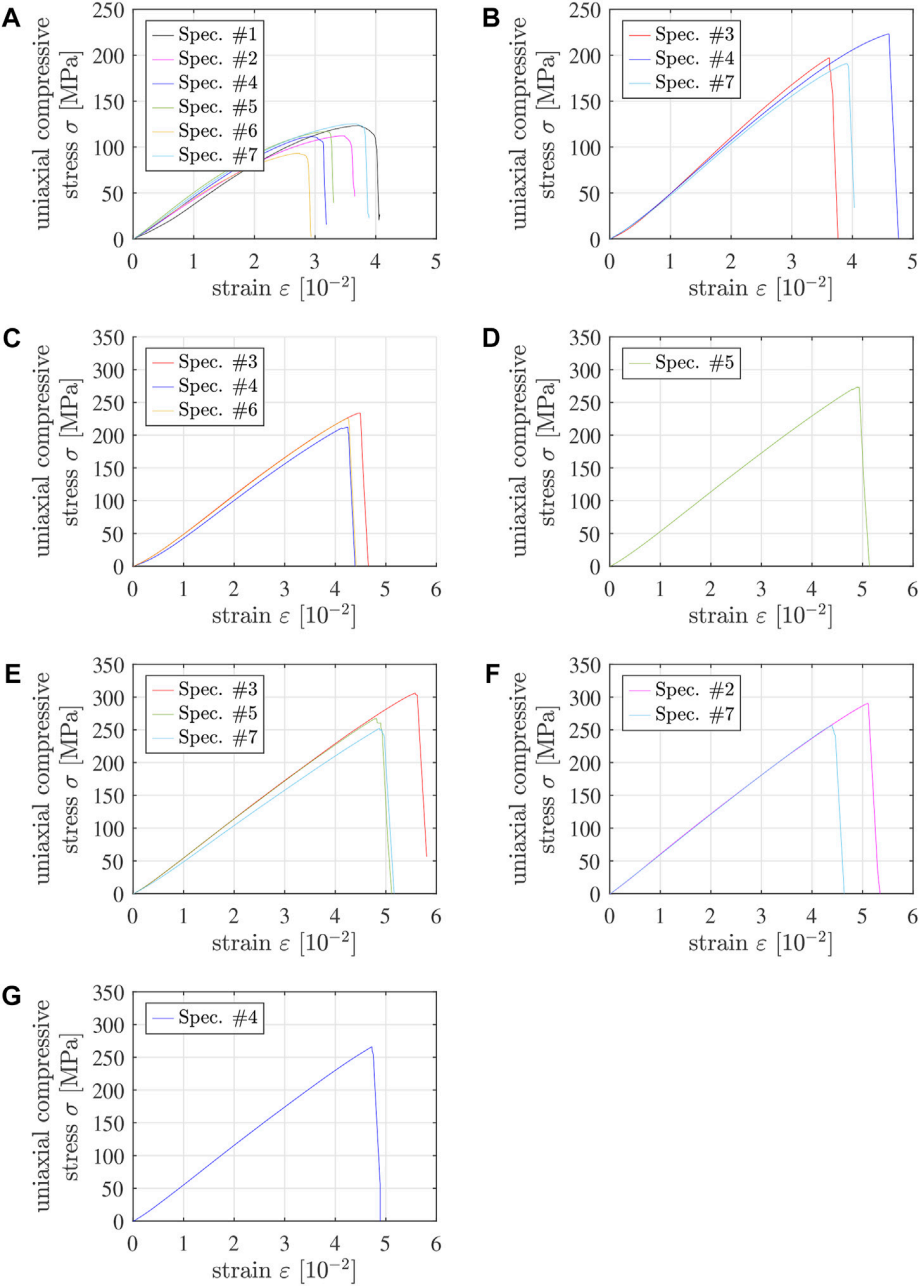
Stress-strain diagrams from destructive compression experiments of 4/6 specimens tested **(A)** 1.5 h, **(B)** 7.5 h, **(C)** 25.5 h, **(D)** 7 days, **(E)** 14 days, **(F)** 21 days, and **(G)** 28 days after production; the solid graphs refer to specimens #1 throughout #7, with the following color code: #1 - black, #2 - pink, #3 - red, #4 - dark blue, #5 - green, #6 - yellow, #7 - light blue, see also Table 5 and Supplementary Table S2. Note that “strain” refers to the sum of the absolute values of the compressive axial strain undergone by the specimen and the tensile axial strain undergone by the testing machine.

Applying the methods described in the preceding two subsections to the Δ*L*(*t*) and *F*(*t*) readings of the testing machine results in 31 stress-strain diagrams of 5/10 specimens and 19 stress-strain diagrams of 4/6 specimens, which are apparently free of significant imperfections, see Figures 4, 5. Qualitative properties of these stress-strain diagrams are discussed in the following two paragraphs.

Some of the graphs exhibit a positive curvature in the first third of the loading interval, see, e.g., the blue graph in Figure 4A. Although this is a sign of geometric imperfections (see above), such tests were nonetheless accepted as long as the stress-strain diagrams increased monotonically all the way up to the strength, without showing pre-peak damage events.

Pre-peak ductility is clearly visible in stress-strain graphs of specimens tested less than 2 h after production, see the non-linearities in the last thirds of the loading intervals in Figures 4A, B as well as in Figure 5A. This ductility decreases with increasing age of Biodentine. One day after production, the material behavior is virtually linear all the way up to sudden brittle failure, see Figures 4E–H, 5C–G. Notably, pre-peak ductility has the potential to compensate for small imperfections regarding coplanarity. Accordingly, the portion of tests discarded because of pre-peak cracking events decreases with decreasing material age, see Tables 2, 3.

### 2.6 Early-age strength evolution

This subsection refers to strength values obtained from stress-strain diagrams of experiments without significant imperfections. The average strength of the 5/10 specimens tested 1 h after production amounts to 50.4 MPa, it almost doubles to 90.9 MPa during the following 30 min, and it virtually doubles again to 203.5 MPa in the subsequent 24 h, see Table 4. Seven days after production, the average strength amounts to 249.7 MPa, 14 days after production to 268.7 MPa, and 21 days after production to 252.2 MPa. All six described mean values were computed from at least three tests which were free of significant imperfections, see Table 4.

**TABLE 4:** Compressive strength values of the 5/10 specimens, from tests which were apparently free of significant imperfections.

Material age	comp. strength *f*_*cu*_ [MPa] of a specimen *n*						
	1	2	3	4	5	6	7
1 h	53.8	50.4	48.0	55.8	−	43.9	−
1.5 h	98.4	69.7	94.0	96.6	82.4	101.8	93.4
3 h	158.2	151.7	−	−	144.2	−	−
7.5 h	155.6	−	162.4	−	−	167.3	−
25.5 h	−	−	−	202.0	209.9	198.6	−
7 days	236.0	274.0	−	−	−	230.1	−
7 days^a^	258.6	−	−	−	−	−	−
14 days	−	−	−	−	−	270.3	275.5
14 days^a^	−	−	−	261.6	−	−	−
21 days	−	261.6	246.5	248.4	−	−	−

^a^ The numbering of the specimens continues as *n* + 7.

The 4/6 specimens show a qualitatively similar early-age strength evolution. The average strength 1.5 h after production amounts to 113.7 MPa, 6 h later it is equal to 203.7 MPa, and 25.5 h after production it amounts to 223.8 MPa, see Table 5. Both 7 and 28 days after production, there is only one test which was apparently free of significant imperfections. 21 days after production there are two such tests. 14 days after production there are three of them, and the average strength amounts to 275.2 MPa, see Table 5.

**TABLE 5:** Compressive strength values of the 4/6 specimens, from tests which were apparently free of significant imperfections.

Material age	comp. strength *f*_*cu*_ [MPa] of a specimen *n*						
	1	2	3	4	5	6	7
1.5 h	123.3	112.2	−	111.4	116.7	93.1	125.2
7.5 h	−	−	197.1	223.1	−	−	190.9
25.5 h	−	−	233.8	212.0	−	225.7	−
7 days	−	−	−	−	273.5	−	−
14 days	−	−	306.1	−	267.4	−	252.2
21 days	−	290.1	−	−	−	−	256.9
28 days	−	−	−	266.0	−	−	−

### 2.7 Failure modes

**FIGURE 6:**
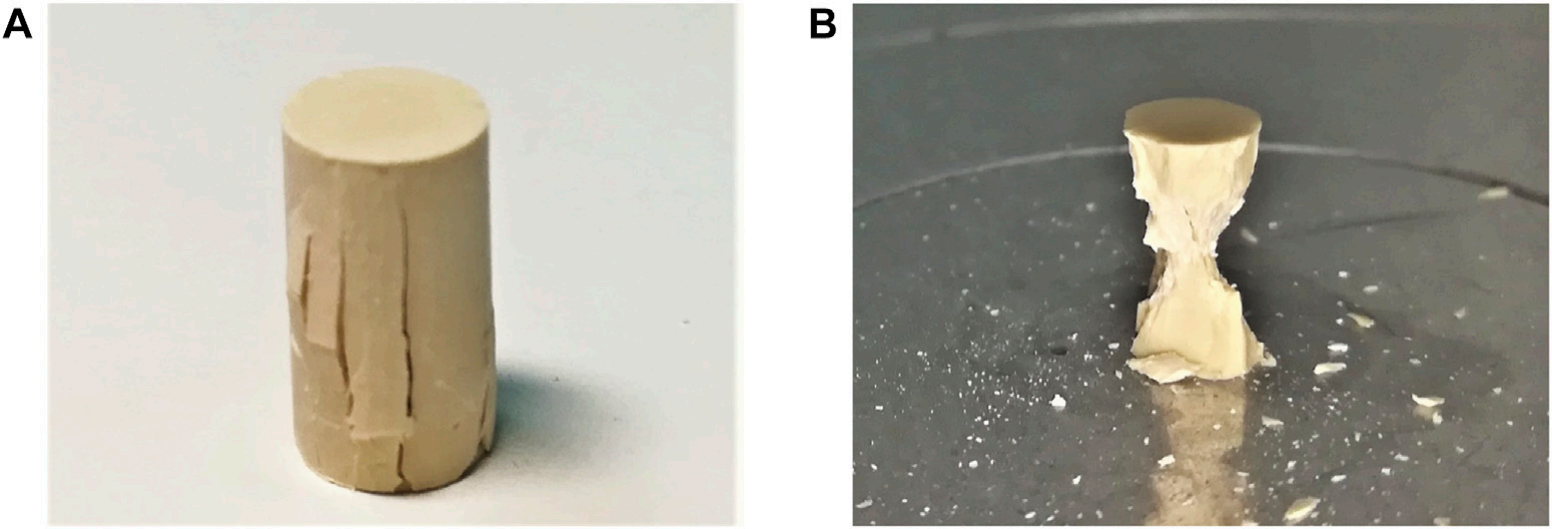
Photos of 5/10 specimens tested at material ages amounting to **(A)** 1 h, and **(B)** 7.5 h.

Both types of specimens exhibited similar failure modes. Axial splitting cracks were observed along the visible lateral surfaces of the specimens, particularly so in the central region of the specimens, i.e., surface cracks were propagating predominantly in loading direction, and only some of them ran across the full height of the specimens, see, e.g., Figure 6A. In several tests, parts of the specimen spalled away during failure from the central region of its lateral surface, leaving behind fragments reminiscent of two cones, whereby the bases of the two cones were located at the interfaces between specimen and the load plates, and the two tips of the cones touched each other at the center of the specimen, see, e.g., Figure 6B. These cones indicate that stresses were fluctuating inside the specimen rather than being uniform throughout the tested volume (Amieur, 1994). These stress fluctuations are known to result from the frictional interaction between the specimen and the load plates (Karte et al., 2015). Notably, the photos of Figure 6 were taken at material ages amounting to 1 h and to 7.5 h, respectively. With increasing maturity, the material became more brittle, and the specimens disintegrated into many pieces. At material ages amounting to 1 day and older, the specimens broke into many fragments, so that classical categorizations concerning the propagation direction of single cracks are not possible anymore.

## 3 Standards-based evaluation of experimental data

### 3.1 Early-age strength evolution

In order to interpolate between the strength values determined by means of testing, and in order to extrapolate to material ages at which no tests were performed, an empirical function is adopted from the *fib* Model Code 2010 (International Federation for Structural Concrete, 2010). Its original version is designed for the description of the early-age strength evolution of construction concretes curing at 20°C, and it reads as

$$f_{c}\left(t\right)=f_{c,28d}\times \exp\left[s\left(1-\sqrt{\frac{28\;days}{t}}\right)\right],$$ (5)

where *f*_*c*_ denotes the compressive strength, *t* the material age (in days), *f*_*c*,28d_ the compressive strength reached 28 days after production, and *s* a dimensionless parameter accounting for the speed of the early-age strength evolution at 20°C. The value of *s* is the smaller the faster the early-age strength evolution, see, e.g., (Ausweger et al., 2019) for a detailed discussion.

The strength of Biodentine curing at 37°C increases significantly up to a material age of 14 days, while it is virtually constant in the third and fourth week after production. This provides the motivation to reformulate Eq. 5 as

$$f_{c}\left(t\right)=f_{c,14d}\times \exp\left[s_{\mathrm{Bio}}\left(1-\sqrt{\frac{14\;days}{t}}\right)\right],$$ (6)

where *f*_*c*,14d_ denotes the compressive strength reached 14 days after production, and *s*_*Bio*_ is a dimensionless parameter accounting for the speed of the early-age strength evolution of Biodentine at 37°C.

The compressive strength reached 14 days after production, *f*_*c*,14d_, is quantified separately for each one of the two types of specimens. Given that the strength was found to be virtually constant throughout the third and fourth week after production, *f*_*c*,14d_ is set equal to the mean value of all strength values obtained 14, 21, and 28 days after production. In both cases, six strength values refer to that interval of material ages, see the last three lines in Tables 4, 5. The corresponding mean values read as

$$5\text{/}10\text{ specimens:}\Rightarrow f_{c,14d}=260.4\;MPa,$$ (7)

$$4\text{/}6\text{ specimens:}\Rightarrow f_{c,14d}=273.1\;MPa,$$ (8)

see also the last line of Table 6 which lists average strength values as a function of specimen type and material age.

**TABLE 6:** Average compressive strength values as a function of specimen type and material age; the underlying individual strength values are listed in Tables 4, 5.

Material age	5/10 specimens		4/6 specimens	
	Mean [MPa]	st. dev. [MPa]	Mean [MPa]	st. dev. [MPa]
1 h	50.4	4.7	−	−
1.5 h	90.9	11.1	113.7	11.5
3 h	151.4	7.0	−	−
7.5 h	161.8	5.9	203.7	17.1
25.5 h	203.5	5.8	223.8	11.0
7 days	249.7	20.3	273.5	−
14–28 days	260.4	10.5	273.1	19.0

**FIGURE 7:**
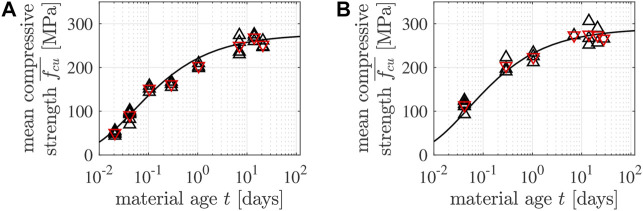
Early-age strength evolution of **(A)** the 5/10 specimens and **(B)** the 4/6 specimens: the black △ symbols mark individual strength values of Tables 4, 5, the red ▽ symbols mark the corresponding mean values of Table 6, and the black solid lines were obtained from the combination of Eqs 6–9.

The early-age strength evolution of both types of specimens is fitted by means of one value of *s*_*Bio*_:

$$s_{\mathrm{Bio}}=0.06,$$ (9)

see the solid lines in Figure 7 for the satisfactory performance of Eqs 6–9 after fitting.

### 3.2 Influence of the length-to-diameter ratio of cylindrical specimens on their compressive strength

**FIGURE 8:**
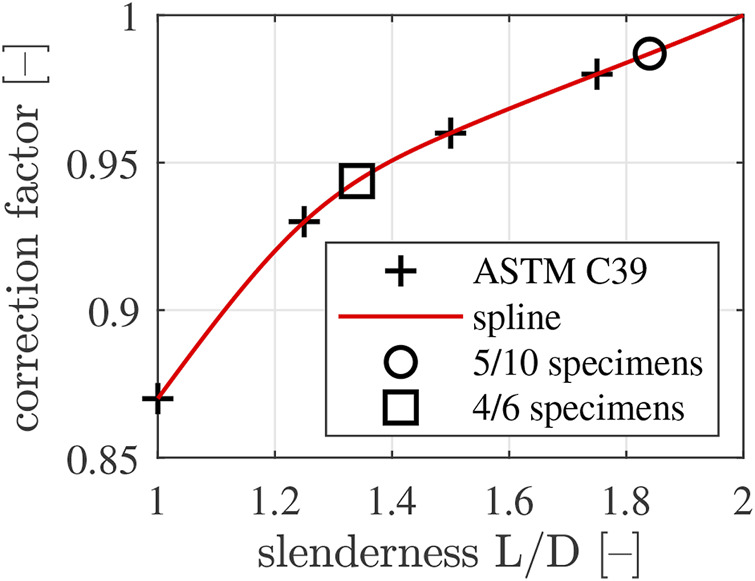
Correction factors compensating the overestimation of the genuine uniaxial compressive strength of concrete as a function of the slenderness-ratio: “+” symbols mark values taken from ASTM C39 standard American Society for Testing and Materials (2021), the red graph is a spline running through the data points of ASTM C39, the “□” symbol marks the correction factor referring to the slenderness-ratio of the 4/6 specimens, and the “◦” symbol marks the correction factor referring to the slenderness-ratio of the 5/10 specimens.

The 5/10 specimens delivered smaller compressive strength values than the 4/6 specimens, throughout the interval of material ages covered by destructive compression tests, see Table 6. This raises the question how to quantify the *genuine uniaxial* compressive strength of Biodentine. It is recalled that the compressive strength of cylinders made of concrete is known to increase with decreasing slenderness-ratio *L*/*D*, where *L* denotes the axial length of the specimen, and *D* its diameter. The ASTM C39 standard (American Society for Testing and Materials, 2021) provides corresponding correction factors which are to be multiplied with experimentally determined compressive strength values, in order to quantify the genuine uniaxial compressive strength, see Figure 8.

In the following, it is checked whether or not the correction factors of ASTM C39 also apply to Biodentine. To this end, the strength evolutions of the two types of specimens, see Eqs 6–9, are multiplied with correction factors taken from Figure 8. A first estimate of the evolution of the genuine uniaxial compressive strength is obtained by inserting the 14-day strength value of the 5/10 specimens according to Eq. 7 together with Eq. 9 into Eq. 6 and by multiplying the resulting expression with the correction factor of the 5/10 specimens (slenderness = 1.84), which amounts to 0.9870, see Figure 8. This yields

$$f_{c}\left(t\right)=257.0\;MPa\times \exp\left[0.06\left(1-\sqrt{\frac{14\;days}{t}}\right)\right].$$ (10)

A second estimate of the evolution of the genuine uniaxial compressive strength is obtained by inserting the 14-day strength value of the 4/6 specimens according to Eq. 8 together with Eq. 9 into Eq. 6 and by multiplying the resulting expression with the correction factor of the 4/6 specimens (slenderness = 1.34), which amounts to 0.9437, see Figure 8. This yields

$$f_{c}\left(t\right)=257.7\;MPa\times \exp\left[0.06\left(1-\sqrt{\frac{14\;days}{t}}\right)\right].$$ (11)

The two independent estimates according to Eqs 10, 11 differ by less than 0.3% at any time *t*. This suggests that the correction factors of ASTM C39 are also applicable to Biodentine. It is concluded that cylindrical specimens with slenderness ratio smaller than two overestimate the genuine uniaxial compressive strength of Biodentine by a factor which is independent of material age. The latter is a function of the slenderness-ratio of the specimen. This underlines that the correction factors of Figure 8 account for a structural (rather than for a material) effect.

The evolution of the genuine uniaxial compressive strength of the material, is set equal to the average value of the right-hand-sides of Eqs 10, 11. This yields

$$f_{c}\left(t\right)=257.4\;MPa\times \exp\left[0.06\left(1-\sqrt{\frac{14\;days}{t}}\right)\right].$$ (12)

Eq. 12 suggest that the strength evolution does, strictly speaking, not come to an end. This is consistent with images of the microstructure of Biodentine taken 4 months after production, which show a significant volume fraction of residual cement grains [see Figure 6 of Dohnalík et al. (2021)], underlining that there is still hydratable matter left. In order to extrapolate the evolution of the uniaxial compressive strength of Biodentine to a material age of 4 months, Eq. 12 is evaluated for *t* = 120 days. This delivers:

$$f_{c}\left(4\;months\right)=257.4\;MPa\times \exp\left[0.06\times \left(1-\sqrt{\frac{14\;days}{120\;days}}\right)\right]=267.8\;MPa.$$ (13)

Notably, at the material age of 4 months, grid nanoindentation tests were performed on Biodentine (Dohnalík et al., 2021).

## 4 Microscopic origin of the macroscopic uniaxial compressive strength of mature Biodentine

This section is devoted to micromechanical modeling of the uniaxial compressive strength of mature Biodentine. In the first two subsections, essential prerequisites are recalled in order to achieve a stand-alone character of this manuscript. Section 4.1 presents results from a grid nanoindentation testing campaign performed on polished surfaces of mature Biodentine, taken from (Dohnalík et al., 2021). Section 4.2 presents a micromechanics model for the elastic stiffness of mature Biodentine, which allows for downscaling macrostresses imposed on a representative volume of mature Biodentine to microstresses experienced by microscopic hydrates, taken from (Dohnalík et al., 2022). The rest of the present Section represents original contributions.

### 4.1 Results from grid nanoindentation on mature Biodentine

In this subsection, essential prerequisites for micromechanical modeling of the uniaxial compressive strength of mature Biodentine are recalled: Results from a grid nanoindentation testing campaign, published in (Dohnalík et al., 2021), are summarized as follows.

**FIGURE 9:**
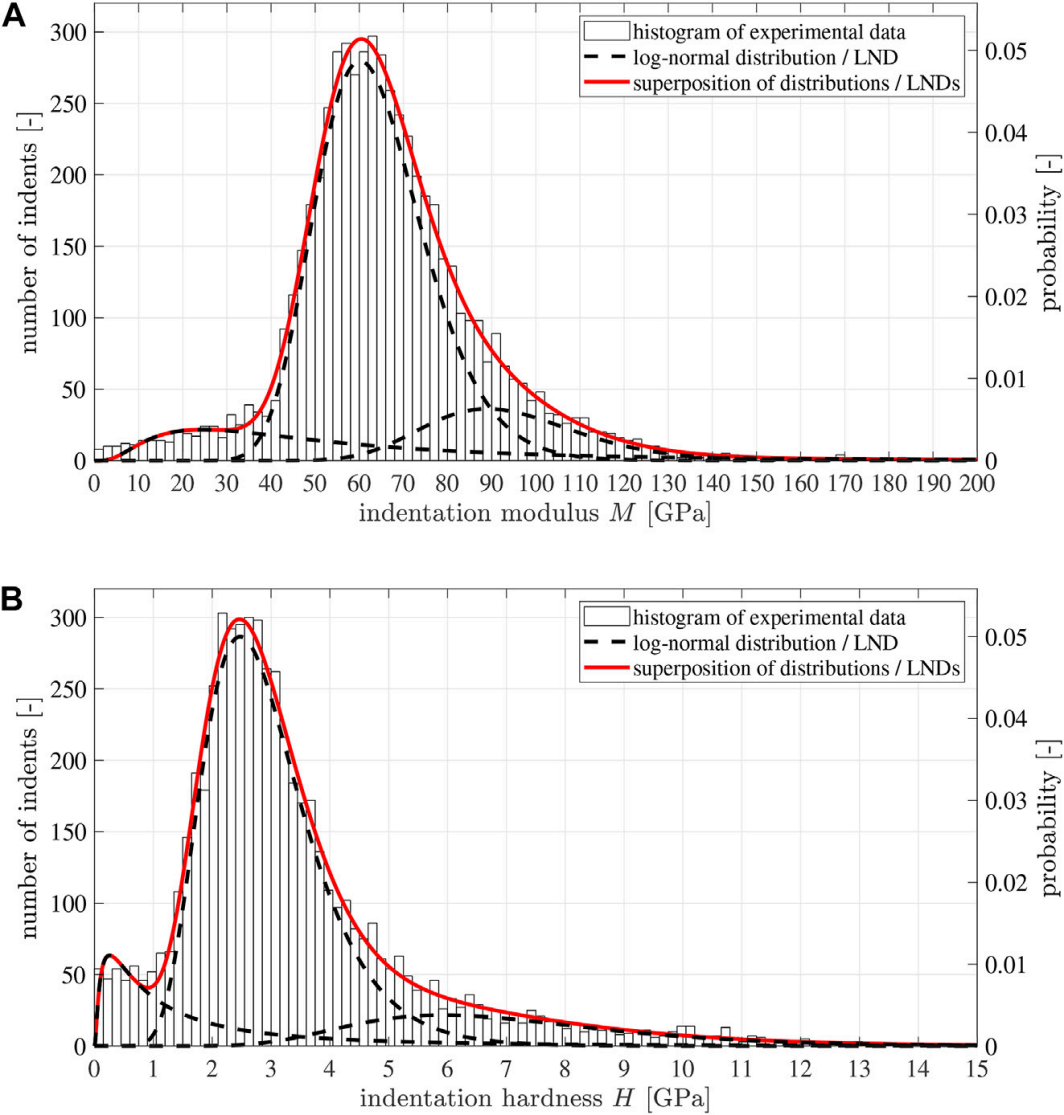
Histograms **(A)** of indentation modulus and **(B)** of the indentation hardness, approximated by means of the superposition of three lognormal distributions; after Dohnalík et al. (2021).

5,748 nanoindentation tests into mature Biodentine were performed with a Berkovich tip (Dohnalík et al., 2021). Imposing maximum indentation forces of 1 mN resulted in maximum indentation depths of on average 140 nm. Only two experiments had to be excluded, because their maximum indentation depths were smaller than 45 nm and, therefore, did not satisfy the requirement of being at least 2.5-times larger than the root-mean-squared average surface roughness (Donnelly et al., 2006; Miller et al., 2008), which amounted to 18 nm (Dohnalík et al., 2021). 5,746 force-displacement diagrams were evaluated based on the Oliver-Pharr formulae for nanoindentation into infinite halfspaces (Oliver and Pharr, 1992). The obtained histograms of the indentation modulus and of the indentation hardness were represented by the superposition of three lognormal probability density functions, see Figure 9. Lognormal distributions were used rather than Gaussians, because i) the indentation moduli are strictly positive quantities, and ii) the large number of indentation experiments revealed skewed rather than symmetric stiffness distributions. The rightmost lognormal distributions in Figure 9 refer to the stiff inclusions zirconia and clinker (indices *i* = 1 and *i* = 2, respectively), the central distributions to high-density calcite-reinforced (HDCR) hydrates (*i* = 3), and the leftmost to lower-density calcite-reinforced (LCDR) hydrates (*i* = 4), see (Dohnalík et al., 2021) and Table 9. This underlines the existence of two types of hydrates reinforced by calcite particles of single-to-submicrometric size (Li et al., 2019). The two populations of hydrates are reminiscent of hardened construction “cement pastes” in which inner and outer products (Taplin, 1959), phenograin and groundmass (Diamond and Bonen, 1993), low-density and high-density C-S-H (Jennings, 2000; Tennis and Jennings, 2000), as well as class-A and class-B C-S-H (Königsberger et al., 2016) are distinguished.

Evaluation of nanoindentation tests into the stiff clinker and zirconia grains, based on the Oliver-Pharr formulae mentioned above, led to smaller-than-expected indentation moduli, because the grains acted like larger indenters pressed into the softer surrounding hydrated material. This effect has been shown explicitly by image-supported grid nanoindentation, applied to two different types of cementitious materials (Ma et al., 2017; Königsberger et al., 2021). Hence, we resort to the well known elastic properties of clinker and zirconia which are taken from the literature, see Table 8. The volume fractions of all four types of solid constituents are taken from (Dohnalík et al., 2021).

The two populations of hydrates exhibit lognormal stiffness distributions, see Table 7. The probability distribution functions of their indentation moduli *M* read as:

$$\varphi_{i}\left(M\right)=\frac{1}{M\sigma_{i}\sqrt{2\pi }}\;\exp\left(-\frac{1}{2}{\left[\frac{\ln\left(M\right)-\mu_{i}}{\sigma_{i}}\right]}^{2}\right),\;i=3,4.$$ (14)

Indentation moduli are functions of the elastic stiffness properties of the nanoindentation-probed domain and of the diamond tip of the indenter. For an isotropic domain, this function reads as (Oliver and Pharr, 1992)

$$\frac{1}{M}=\frac{1-\nu_{h}^{2}}{E}+\frac{1-0.07^{2}}{1141\;GPa},$$ (15)

where *E* and *ν*_*h*_ denote the modulus of elasticity and Poisson’s ratio of the hydrates. The latter is constant and amounts to (Dohnalík et al., 2022)

$$\nu_{h}=0.2017.$$ (16)

In other words, the distribution of indentation modulus is related to a corresponding distribution of the modulus of elasticity, *E*. The latter distribution follows from solving Eq. 15 for *E*:

$$E\left(M\right)=\frac{1-\nu_{h}^{2}}{\frac{1}{M}-\frac{1-0.07^{2}}{1141\;GPa}}.$$ (17)

The sought bulk moduli *k*_*i*_(*M*) and the shear moduli *g*_*i*_(*M*) follow from standard relations for isotropic elastic media:

$$k_{i}\left(M\right)=\frac{E\left(M\right)}{3\;\left(1-2\;\nu_{h}\right)},\;i=3,4,$$ (18)

$$g_{i}\left(M\right)=\frac{E\left(M\right)}{2\;\left(1+\nu_{h}\right)},\;i=3,4.$$ (19)

**TABLE 7:** Results obtained from grid nanoindentation testing (Dohnalík et al., 2021): values of medians, modes, and volume fractions associated with the three lognormal distributions representing the histogram of indentation moduli in Figure 9.

Lognormal	Indentation modulus	Indentation hardness	Volume
distribution	median/mode [GPa]	median/mode [GPa]	fraction [–]
LDCR hydrates	45.1/24.5	1.15/0.26	0.1228
HDCR hydrates	62.6/60.2	2.78/2.47	0.7420
clinker/zirconia	92.2/89.0	6.66/5.93	0.1352
		sum:	1.0000

### 4.2 Continuum micromechanics model for the elastic stiffness of mature Biodentine

In this subsection, essential prerequisites for micromechanical modeling of the uniaxial compressive strength of mature Biodentine are recalled: A micromechanics model for the elastic stiffness of mature Biodentine, which allows for downscaling macrostresses imposed on a representative volume of mature Biodentine to microstresses experienced by microscopic hydrates, published in (Dohnalík et al., 2022), is summarized as follows.

Continuum micromechanics models account for four key features of microheterogeneous materials, rather than for every detail of their microstructures: i) the characteristic shapes of the material phases, ii) the specific type of interaction between the phases, iii) their stiffness constants, and iv) their volume fractions. Herein, the microstructural representation of Biodentine is taken over from (Dohnalík et al., 2022). According to that, Biodentine consists of five types of constituents: zirconia (index *i* = 1), clinker (*i* = 2), HDCR hydrates (*i* = 3), LDCR hydrates (*i* = 4), and grain boundary defects modeled as closed microcracks (*i* = 5). The *four* key features accounted for by the here-used continuum micromechanics model, are discussed, one by one, in the following paragraphs.

**FIGURE 10:**
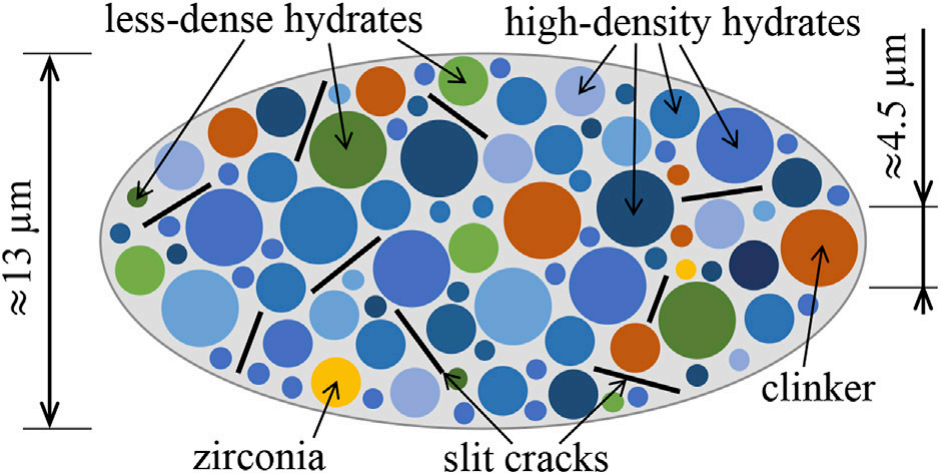
Continuum micromechanics representation of Biodentine after Dohnalík et al. (2022): spherical solid phases and isotropically oriented circular slit cracks in direct mechanical interaction.

i) Characteristic shapes of the material phases: Light microscopy images of polished surfaces of mature Biodentine show virtually spherical clinker and zirconia grains, see Figures 5, 6 of (Dohnalík et al., 2021). It is reasonable to assign spherical phase shapes also to the hydrates, because potentially non-spherical phase shapes of solid constituents are important for homogenization of highly porous microstructures, while they play an insignificant role for homogenization of dense microstructures (Pichler et al., 2009), such as the here-analyzed microstructure of mature Biodentine. Thus, all four types of solid constituents are represented as spherical phases, see Figure 10 for a two-dimensional sketch illustrating *qualitative* features of the three-dimensional representative volume elements of Biodentine. The microcracks are introduced as circular slit cracks isotropically oriented in space (Dormieux et al., 2006; Zhu et al., 2011).

ii) Interaction between the material phases: The constituents of Biodentine exhibit *direct* phase-to-phase interaction (Dohnalík et al., 2022). Therefore, the self-consistent scheme of continuum micromechanics (Zaoui, 2002; Bernard et al., 2003; Dormieux et al., 2006) will be used for scale transitions, i.e., for upscaling of the elastic stiffness and for downscaling of stresses. This mean-field method provides quantitative access to volume-averaged stresses experienced by the material phases (rather than resolving microscopic stress fluctuations in full detail), see Supplementary Section S3.

iii) Stiffness constants of the material phases: The stiffness of all solid constituents is isotropic. Their elastic stiffness properties are fully described based on known values of their bulk moduli *k*_*i*_ and shear moduli *g*_*i*_, see Table 8 as well as Eqs 14–19.

**TABLE 8:** Input quantities of the solid material constituents: bulk moduli, *k*_*i*_, shear moduli *g*_*i*_, as well as lognormal parameters *μ*_*i*_ and *σ*_*i*_ which are consistent with median and mode values listed in Table 7; input values taken from (Hussey and Wilson, 1998), (Pichler and Hellmich, 2011), and (Dohnalík et al., 2021).

Phase	Index	Stiffness properties		vol. fraction
zirconia	*i* = 1	*k*_1_ = 170.8 GPa	*g*_1_ = 78.8 GPa	*f*_1_ = 0.0182
clinker	*i* = 2	*k*_2_ = 116.7 GPa	*g*_2_ = 53.8 GPa	*f*_2_ = 0.1170
HDCR hydrates	*i* = 3	*μ*_3_ = 4.14	*σ*_3_ = 0.20	*f*_3_ = 0.7420
LDCR hydrates	*i* = 4	*μ*_4_ = 3.81	*σ*_4_ = 0.78	*f*_4_ = 0.1228

iv) Volume fractions of the material phases: Phase volume fractions of the solid constituents add up to 1, see Table 8, because the volume fraction of closed microcracks is zero. The effect of such microcracks on the overall material behavior is quantified by means of the dimensionless crack density parameter of Budiansky and O’Connell (1976), see also (Deudé et al., 2002; Pensée et al., 2002). As for Biodentine, the crack density parameter was identified by Dohnalík et al. (2022) such that the model reproduces the actual isotropic macrostiffness of mature Biodentine. The corresponding bulk and shear moduli of mature Biodentine were quantified from ultrasonic pulse velocity measurements using both longitudinal and transversal waves with frequencies in the interval from 50 kHz to 20 MHz. The crack density parameter amounts to:

ω=0.7802. (20)

Interfaces between microstructural constituents were considered as follows. Perfect bond along all interfaces is simulated by the used self-consistent homogenization scheme, provided that it is applied to microstructures consisting of solid phases only. The microstructure of Biodentine, in turn, contains weakening zero-volume interfaces, because a *lower* stiffness bound computed under consideration of a perfectly bonded solid microstructure was found to be *larger than* the homogenized stiffness quantified by means of ultrasound characterization (Dohnalík et al., 2021). Therefore, the representation of Biodentine was extended towards consideration of isotropically oriented, closed, circular slit cracks (Dohnalík et al., 2022). Under macroscopic loading of Biodentine, these microcracks develop shear slip, and this models shear dislocations between the solid material phases. Thus, the microcracks are to be understood as a continuum micromechanics approach to weak interfaces between neighboring solid microstructural constituents.

Stress downscaling, from the macroscopic stress **Σ** imposed on a representative volume element of mature Biodentine, down to microscopic stresses of the two populations of hydrates is based on stress downscaling tensors $B_{3}\left(M\right)$ and $B_{4}\left(M\right)$

$$\sigma_{i}\left(M\right)=B_{i}\left(M\right):\Sigma ,\;i=3,4.$$ (21)

The stress downscaling tensors are isotropic

$$B_{i}\left(M\right)=B_{\mathrm{vol,i}}\left(M\right)\;I^{\mathrm{vol}}+B_{\mathrm{dev,i}}\left(M\right)\;I^{\mathrm{dev}},\;i=3,4,$$ (22)

where $I^{\mathrm{vol}}$ and $I^{\mathrm{dev}}$ stand for the volumetric and deviatoric parts of the symmetric fourth-order identity tensor I . Their components read as $I_{\mathrm{ijkl}}=\left(\delta_{ik}\delta_{jl}+\delta_{il}\delta_{jk}\right)/2$ , $I_{\mathrm{ijkl}}^{\mathrm{vol}}=\left(\delta_{ij}\delta_{kl}\right)/3$ , and $I_{\mathrm{ijkl}}^{\mathrm{dev}}=I_{\mathrm{ijkl}}-I_{\mathrm{ijkl}}^{\mathrm{vol}}$ , where *δ*_*ij*_ is the Kronecker delta which is equal to 1 for *i* = *j*, and equal to 0 otherwise. The volumetric and deviatoric components of the stress downscaling tensors, *B*_*vol,i*_(*M*) and *B*_*dev,i*_(*M*), respectively, read as (Dohnalík et al., 2022).

$$\begin{array}{ccc} B_{\mathrm{vol,i}}\left(M\right) & = & \frac{k_{i}\left(M\right)/k_{\mathrm{bio}}}{1+\frac{S_{\mathrm{vol}}\left(k_{i}\left(M\right)-k_{\mathrm{bio}}\right)}{k_{\mathrm{bio}}}}\{\overset{2}{\underset{i=1}{\sum }}\frac{f_{i}}{1+\frac{S_{\mathrm{vol}}\left(k_{i}-k_{\mathrm{bio}}\right)}{k_{\mathrm{bio}}}}\\ & & {+\overset{4}{\underset{i=3}{\sum }}f_{i}\int_{0}^{\infty }\frac{\varphi_{i}\left(M\right)}{1+\frac{S_{\mathrm{vol}}\left(k_{i}\left(M\right)-k_{\mathrm{bio}}\right)}{k_{\mathrm{bio}}}}\;dM\}}^{-1}, \end{array}$$ (23)

$$\begin{array}{ccc} B_{\mathrm{dev,i}}\left(M\right) & = & \frac{g_{i}\left(M\right)/g_{\mathrm{bio}}}{1+\frac{S_{\mathrm{dev}}\left(g_{i}\left(M\right)-g_{\mathrm{bio}}\right)}{g_{\mathrm{bio}}}}\{\overset{2}{\underset{i=1}{\sum }}\frac{f_{i}}{1+\frac{S_{\mathrm{dev}}\left(g_{i}-g_{\mathrm{bio}}\right)}{g_{\mathrm{bio}}}}\\ & & {\left.+\overset{4}{\underset{i=3}{\sum }}f_{i}\int_{0}^{\infty }\frac{\varphi_{i}\left(M\right)}{1+\frac{S_{\mathrm{dev}}\left(g_{i}\left(M\right)-g_{\mathrm{bio}}\right)}{g_{\mathrm{bio}}}}\;dM+\frac{4\pi \omega }{3}\;T_{\mathrm{dev}}\right\}}^{-1}, \end{array}$$ (24)

where *i* = 3, 4 and $M\in R^{0,+}$ and

$$S_{\mathrm{vol}}=\frac{3k_{\mathrm{bio}}}{3k_{\mathrm{bio}}+4g_{\mathrm{bio}}},$$ (25)

$$S_{\mathrm{dev}}=\frac{6\left(k_{\mathrm{bio}}+2g_{\mathrm{bio}}\right)}{5\left(3k_{\mathrm{bio}}+4g_{\mathrm{bio}}\right)},$$ (26)

$$T_{\mathrm{dev}}=\frac{8\;\left(3k_{\mathrm{bio}}+4g_{\mathrm{bio}}\right)}{15\pi \;\left(3k_{\mathrm{bio}}+2g_{\mathrm{bio}}\right)},$$ (27)

$$k_{\mathrm{bio}}=38.4\;GPa,$$ (28)

$$g_{\mathrm{bio}}=14.1\;GPa,$$ (29)

where *S*_*vol*_ and *S*_*dev*_ denote the volumetric and deviatoric components of the Eshelby tensor of a spherical inclusion in an infinite matrix with the stiffness of mature Biodentine, *T*_*dev*_ accounts for the influence of the microcracks, and *k*_*bio*_ and *g*_*bio*_ are the macroscopic “homogenized” bulk and shear moduli of mature Biodentine (Dohnalík et al., 2022). The integrals of Eqs 23, 24 are listed in (Dohnalík et al. 2022), more precisely in Appendix B of the aforementioned paper.

Finally, it is worth emphasising that Figure 10 shows qualitative features of the highly disordered microstructure of mature Biodentine (rather than a Finite Element discretization). These features are accounted for by means of the employed self-consistent scheme of continuum-micromechanics. In this context, it is noteworthy that the two populations of hydrates are represented as two times infinitely many material phases. This investment allows us i) to account for the *continuous* stiffness distributions of both populations of hydrates, and ii) to compute, for every specific stiffness, corresponding volume-averaged microstresses resulting from a specific macroscopic state of loading imposed on a representative volume of mature Biodentine. In other words, we will be able to compute continuous stress distributions experienced by the two populations of hydrates. Still, details on the spatial arrangement of the microstructural constituents of mature Biodentine remain unresolved, see Supplementary Section S3 for more information on this aspect.

### 4.3 Microscopic stress distributions in calcite-reinforced hydrates resulting from macroscopic uniaxial compressive loading

In order to explain the microscopic origin of the macroscopic uniaxial compressive strength of mature Biodentine according to Eq. 13, a Cartesian coordinate system is introduced such that the direction of uniaxial compression is aligned with the *z*-direction. Thus, the stress state at failure is expressed as^2^

$$\Sigma =-267.8\;MPa\times \left(e_{z}⊗e_{z}\right).$$ (30)

Notably, Eq. 30 does not imply that the stress state in a cylindrical sample with length-to-diameter ratio = 2.0 is uniform. This is explained next. A uniaxial compression test requires loading by means of uniform normal tractions. However, friction in the interfaces between the load plates and the specimen activates also shear tractions. According to the principle of St. Venant (Barré Saint-Venant, 1855), these undesired shear stresses decrease inside the specimen with increasing distance from the load plate. They are only significant up to a distance from the load plate, which is similar to the characteristic size of the contact area between load plate and the specimen, see Figure 2 of (Karte et al., 2015). This implies that the central region of a cylindrical sample with length-to-diameter ratio equal to 2, is virtually free of undesired shear stresses. In this region, a virtually uniform uniaxial compressive stress state prevails. Failure of the specimen originates from this region. The following part of the study makes use of this effect. It is focused on a representative volume of mature Biodentine, which is located in such a central region free of shear stresses, such that the stress state at failure reads as the one given in Eq. 30.

**FIGURE 11:**
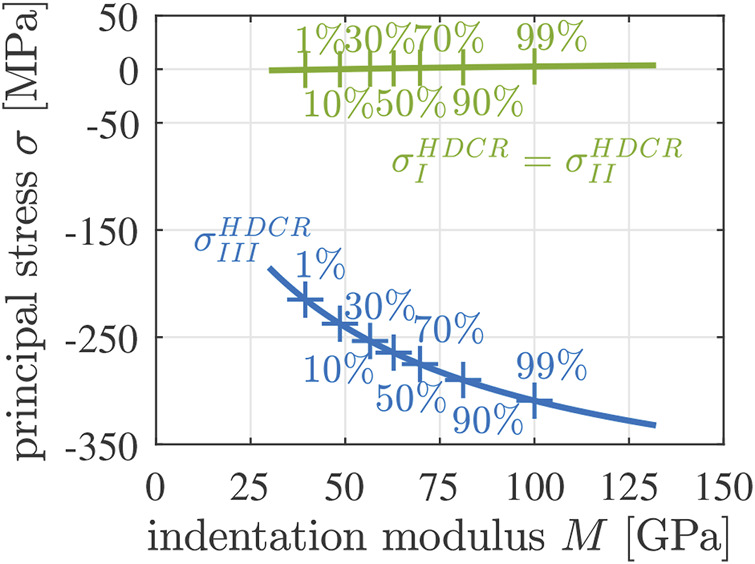
Axial (blue) and lateral (green) components of the microscopic principal stress states experienced by the HDCR population of hydrates, as a function of the indentation modulus *M*; standard sign convention of continuum mechanics: compression is negative, tension is positive.

Downscaling of the stress state given in Eq. 30, according to Eqs 21–29, delivers axisymmetric microscopic principal stress states experienced by the two populations of hydrates. Denoting the normal stress component in the axial, i.e. *z*-direction as *σ*_*axi*_ and the normal stress components in the laterial, i.e. *x*- and *y*-directions as *σ*_*lat*_, the microscopic stress states read as

$$\sigma_{i}\left(M\right)=\sigma_{i,axi}\left(M\right)\times \left(e_{z}⊗e_{z}\right)+\sigma_{i,lat}\left(M\right)\times \left(e_{x}⊗e_{x}+e_{y}⊗e_{y}\right),i=3,4,\left(31\right)$$ (31)

see also Figure 11. The absolute values of the compressive axial normal stresses increase with increasing indentation modulus of the HDCR hydrates. The lateral principal normal stresses are negligibly small compared to the axial normal stresses.

### 4.4 Shear failure of calcite-reinforced hydrates

Cementitious hydration products resulting from the dissolution of dicalcium and tricalcium silicate and from precipitation of solids out of the oversaturated porewater solution, are known to exhibit pressure-sensitive shear failure (Constantinides et al., 2006; Pichler et al., 2013b; Sarris and Constantinides, 2013; Königsberger et al., 2018). The corresponding Mohr-Coulomb failure criterion reads as (Königsberger et al., 2018):

$$f\left(\sigma \right)=\sigma_{I}\;\frac{1+\sin\varphi }{2c\;\cos\varphi }-\sigma_{\mathrm{III}}\;\frac{1-\sin\varphi }{2c\;\cos\varphi }\leq 1,$$ (32)

where *σ*_*I*_ denotes the largest principal normal stress, *σ*_*III*_ the smallest principal normal stress, *c* the cohesion, and *φ* the angle of internal friction. *f*(***σ***) < 0 refers to stress states sustained by the material by means of linear elastic behavior, while *f*(***σ***) = 0 refers to brittle failure. Recalling that *σ*_*lat*_ = *σ*_*I*_ is negligibly small compared to *σ*_*axi*_ = *σ*_*III*_ < 0 and introducing an equivalent shear strength *C* such that 1/(2*C*) is equal to the term multiplied by *σ*_*III*_ in Eq. 32,

$$C=c\;\frac{\cos\varphi }{1-\sin\varphi },$$ (33)

the failure criterion according to Eq. 32 can be rewritten as

$$\frac{\left|\sigma_{\mathrm{axi}}\right|}{2}\leq C,$$ (34)

where the < sign refers to linear elastic behavior and the = sign to brittle failure. Notably, |*σ*_*axi*_| increases with increasing stiffness of the hydrates, see Figure 11. Given that the indentation modulus and the indentation hardness of the hydrates are lognormally distributed, also the equivalent shear strength is modeled as a lognormally distributed quantity. This provides the motivation to establish correlations between lognormal distributions of indentation modulus, indentation hardness, and the equivalent shear strength.

### 4.5 Correlations between lognormal distributions of indentation modulus, indentation hardness, and the equivalent shear strength

Correlations between lognormal distributions of the indentation modulus *M*, the indentation hardness *H*, and the equivalent shear strength *C* are established by relating all *p*-quantiles of any lognormal distribution to the same *p*-quantiles of the other two lognormal distributions. To this end, we introduce the *p*-quantiles of two lognormal distributions referring to statistical variables *X* and *Y* as

$$X_{p}=\exp\left(\mu_{X}+u_{p}\sigma_{X}\right),$$ (35)

$$Y_{p}=\exp\left(\mu_{Y}+u_{p}\sigma_{Y}\right),$$ (36)

with *X* ∈ [*M*, *H*, *C*] and *Y* ∈ [*M*, *H*, *C*]. In Eqs 35, 36, *σ*_*X*_ and *μ*_*X*_ as well as *σ*_*Y*_ and *μ*_*Y*_ denote the parameters of the two lognormal distributions, and *u*_*p*_ stands for the *p*-quantile of the standard normal distribution. Solving both Eqs 35, 36 for *u*_*p*_, setting the two resulting expressions equal to each other, and transforming the resulting expression as detailed in Appendix A, yields the following remarkable result: relating any *p*-quantile of one lognormally distributed statistical variable *X* to *the same* *p*-quantile of another lognormally distributed statistical variable *Y* leads to the following power-law relation between the two statistical variables:

$$Y=\exp\left(\mu_{Y}-\frac{\sigma_{Y}}{\sigma_{X}}\;\mu_{X}\right)\times X^{\frac{\sigma_{Y}}{\sigma_{X}}}.$$ (37)

**FIGURE 12:**
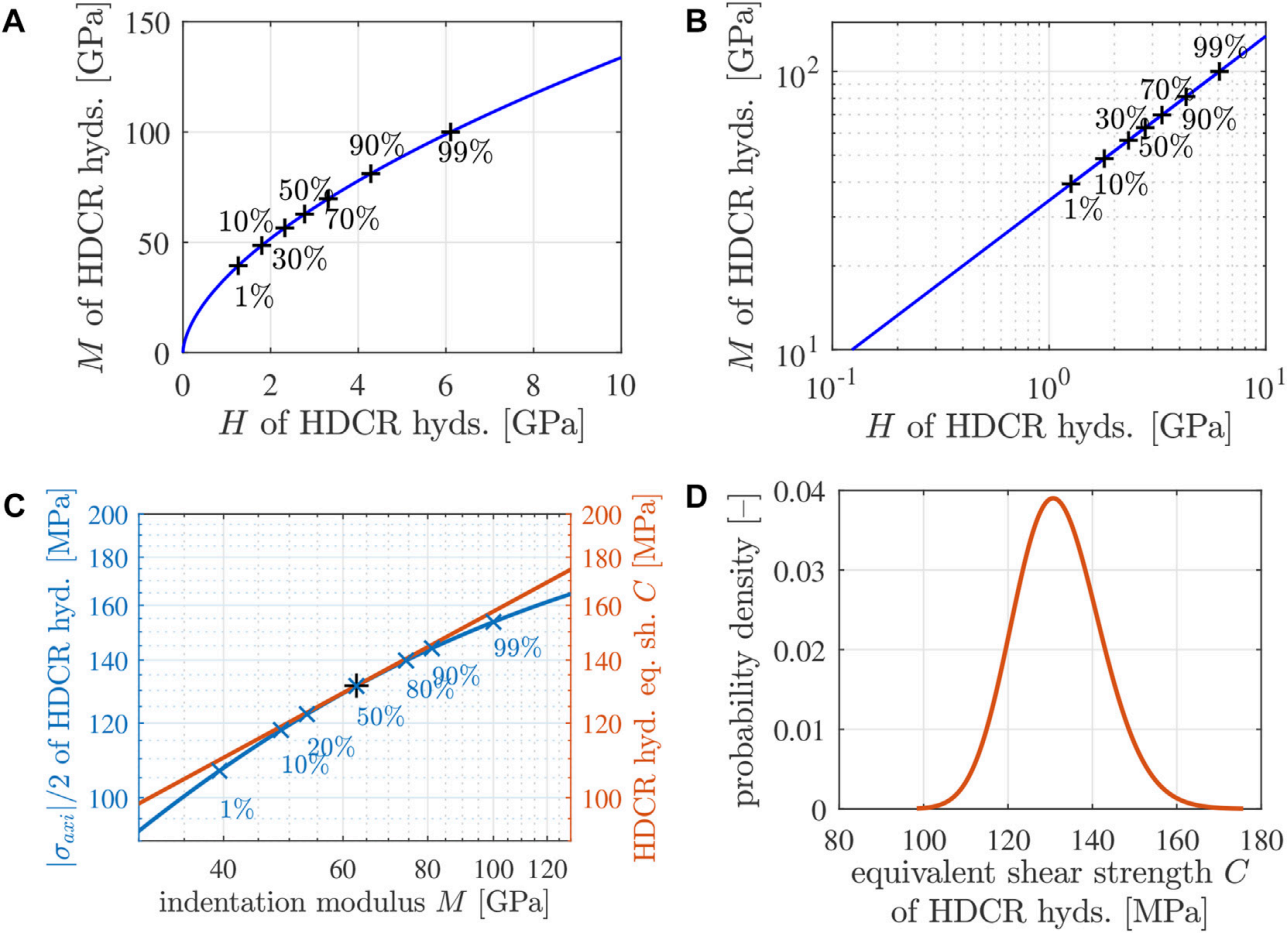
**(A, B)** Quantile-based correlation between the lognormal distributions of the indentation modulus *M* and the indentation hardness *H* of the HDCR hydrates: representation of the power-law-relation according to Eq. 37 with *X* = *H*, *Y* = *M*, *μ*_*M*_ = 4.14, *σ*_*M*_ = 0.20, *μ*_*H*_ = 1.02, and *σ*_*H*_ =0.34, see Table 8 and (Dohnalík et al., 2021): **(A)** natural scale, **(B)** double-logarithmic representation; the black “+” symbols mark *p*-quantiles, with values of *p* equal to the printed percentage values. **(C)** Illustration of the failure criterion of Eq. 34: the equivalent shear strength of all HDCR hydrates (orange line) is larger than |*σ*_*axi*_/2| (blue line), except for the median value of the indentation modulus, for which |*σ*_*axi*_/2| = *C*; the blue “+” symbols mark *p*-quantiles, with values of *p* given as percentage values. **(D)** Lognormal probability density distribution of the equivalent shear strength of the HDCR hydrates explaining macroscopic failure of mature Biodentine under uniaxial compression according to Eq. 30; the lognormal parameters are given in Eqs 39, 40.

Setting *X* equal to the indentation hardness *H* as well as *Y* equal to the indentation modulus *M*, yields a power-law describing that the stiffness increases underlinearly with increasing strength, see Figures 12A, B. This is qualitatively reminiscent of similar power-law relations between stiffness and strength of mature construction concretes, see the *fib* Model Code 2010 (International Federation for Structural Concrete, 2010) and Figure 6 of (Ausweger et al., 2019).

### 4.6 Identification of lognormal parameters of the equivalent shear strength

The microscopic stresses experienced by the hydrates, see Figure 11, were obtained from downscaling the macroscopic uniaxial compressive *strength* of mature Biodentine. Thus, one specific portion of the HDCR hydrates (with one specific value of the indentation modulus) must fulfill the failure criterion of Eq. 34, consider the = sign in Eq. 34, while the < sign applies to all other hydrates. This represents a side condition for the sought lognormal distribution of the equivalent shear strength.

As for identification of the lognormal distribution of the equivalent shear strength of the HDCR hydrates, is useful to illustrate their stress states as a function of their indentation modulus in a double logarithmic fashion, see Figure 12C, where the ordinate refers to |*σ*_*axi*_/2| rather than to |*σ*_*axi*_|, because the figure will be used to illustrate the failure criterion of Eq. 34.

The *sought* lognormal distribution of the equivalent shear strength is related to the *known* lognormal distribution of the indentation modulus by correlating their quantiles as explained in Section 4.5. The resulting power-law is obtained from setting *X* equal to *M* and *Y* equal to *C* in Eq. 37:

$$C=\exp\left(\mu_{C}-\frac{\sigma_{C}}{\sigma_{M}}\;\mu_{M}\right)\times M^{\frac{\sigma_{C}}{\sigma_{M}}},$$ (38)

where *μ*_*M*_ = 4.14 and *σ*_*M*_ = 0.20, see Table 8, while *μ*_*C*_ and *σ*_*C*_ are to be identified. As for adding a graphical illustration of Eq. 38 to Figure 12C a second ordinate showing *C* is added. The power-law Eq. 38 refers to a straight line in Figure 12C. This straight line can be moved and rotated in Figure 12C, by means of assigning different values to *μ*_*C*_ and *σ*_*C*_. A realistic pair of values is identified such that the straight line becomes the tangent to the graph showing |*σ*_*axi*_/2| over *M*. The contact point refers to those hydrates which fulfill the failure criterion of Eq. 34. Given that median stiffness values have been shown to be the most representative values of lognormal distributions when it comes to upscaling of the elastic stiffness (Dohnalík et al., 2022), the microstresses experienced by the hydrates with the median stiffness are taken as relevant for strength upscaling. In other words, *μ*_*C*_ and *σ*_*C*_ are identified such that the graph of Eq. 38 touches the graph of the axial stresses at the median value of *M*, i.e., at the 50% quantile of *M*, amounting to 62.6 GPa for the studied HDCR hydrates, see Figure 12C and Table 7. The corresponding lognormal parameters of the distributions of the equivalent shear strength of the HDCR hydrates read as

$$\mu_{C}=4.879,$$ (39)

$$\sigma_{C}=0.078.$$ (40)

The corresponding probability density function of the equivalent shear strength is illustrated in Figure 12D. Corresponding values of the mode, the median, and the mean value are listed in Table 9. The lognormal distribution of the effective shear strength is only slightly skewed. This is in agreement with the only slightly skewed distributions of the indentation modulus and hardness of the HDCR hydrates.

**TABLE 9:** Mode, median, and mean value of the lognormal distribution of the equivalent shear strength *C* of the HDCR hydrates, see also Eqs 39, 40.

Mode [MPa]	Median [MPa]	Mean [MPa]
130.7	131.5	131.9

### 4.7 Degree of utilization of hydrates as a function of their indentation moduli

**FIGURE 13:**
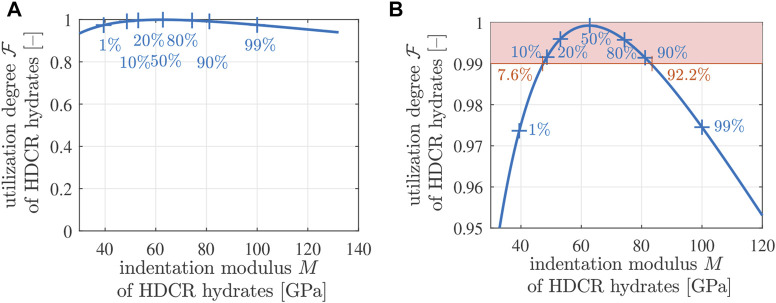
Degree of utilization $F=|\sigma_{\mathrm{axi}}|/\left(2C\right)$ as a function of the indentation modulus *M* for HDCR hydrates; the “+” symbols mark *p*-quantiles, with values of *p* given as percentage values: **(A)** overview, **(B)** detail.

The degree of utilization is a dimensionless stress-based quantity ranging from 0 to 1. The value 0 refers to a stress-free configuration; the value 1 to failure. The larger the degree of utilization, the closer is the investigated stress state to the strength of the analyzed material. A mathematical expression describing the degree of utilization F is obtained from dividing the failure criterion of Eq. 34 by *C*

$$F=\frac{\left|\sigma_{\mathrm{axi}}\right|}{2C}\leq 1,$$ (41)

where both the microstresses *σ*_*axi*_ and the equivalent shear strength *C* are functions of the indentation modulus, see Figure 12C. The corresponding evaluation of Eq. 41 underlines that the maximum attainable degree of utilization, F=1 , is only reached by the hydrates with indentation modulus equal to the median value, while all other hydrates have degrees of utilization smaller than 1, see Figure 13A. Still, it is remarkable that the degree of utilization of all HDCR hydrates is larger than some 93%. This underlines that mature Biodentine is a highly optimized material.

### 4.8 Amount of HDCR hydrates with degree of utilization F≥99%

Mature Biodentine fails in a brittle fashion. Corresponding specimens break into a very high number of fragments once the strength is reached. Therefore, it is likely that many hydrates fail at virtually the same macroscopic stress intensity. This provides the motivation to quantify the amount of hydrates which have a degree of utilization of 99% or larger. All HDCR hydrates between the 7.6%-quantile and the 92.2%-quantile have degrees of utilization larger than or equal to 99%, see Figure 13B. In other words, 92.2% − 7.6% = 84.6% of the HDCR hydrates have a degree of utilization larger than or equal to 99%. Therefore, they will fail virtually simultaneously. Given that HDCR hydrates make up 74.20% of the volume of Biodentine, see Table 8, 84.6 % × 74.20 % = 62.8% of the volume of Biodentine fails at the same time. This is consistent with destructive mechanical testing having the Biodentine samples broken into many fragments.

The presented analysis was carried out under the assumption that the HDCR population of hydrates is responsible for failure of mature Biodentine. Repeating the analysis under the alternative assumption that the LDCR population of hydrates triggers failure of mature Biodentine, see Supplementary Section S2, leads to the results that only 3.5% of the volume of Biodentine would fail virtually at the same time, but that would lead to pre-peak non-linearities rather than to the experimentally observed sudden brittle mode of failure associated with many fragments.

## 5 Discussion

This section is devoted to the discussion of the storage conditions, a comparison of the actual elastic stiffness of mature Biodentine with the slope of stress-strain diagrams from the compressive strength tests, and similarities and differences between construction “cement pastes” and Biodentine.

### 5.1 Storage conditions

Early-age drying of specimens made from cementitious materials leads to gradients of internal relative humidity, to shrinkage strains which are restraint, and, therefore, to cracking of the specimens initially perpendicular to the drying surface, and later parallel to the drying surfaces (Shiotani et al., 2003). Storage of cementitious materials in distilled water avoids drying, but leads to decalcification, increase of porosity, and loss of strength (Carde and Francois, 1999). Storing the here-tested specimens submersed in lime-saturated solution achieved its purpose of avoiding both drying of the specimens and leaching of calcium from their microstructures. It is noted that the lime-saturated solution might have interacted with Biodentine, affecting its microstructure potentially in an adverse fashion. However, this goes beyond the scope of the present paper and provides the motivation for future studies. It will be also interesting to study the durability of calcite-reinforced hydrates under *in vivo* conditions and how this affects the resultant volume of mature Biodentine.

### 5.2 Actual elastic stiffness of mature Biodentine vs. slope of stress-strain diagrams

**FIGURE 14:**
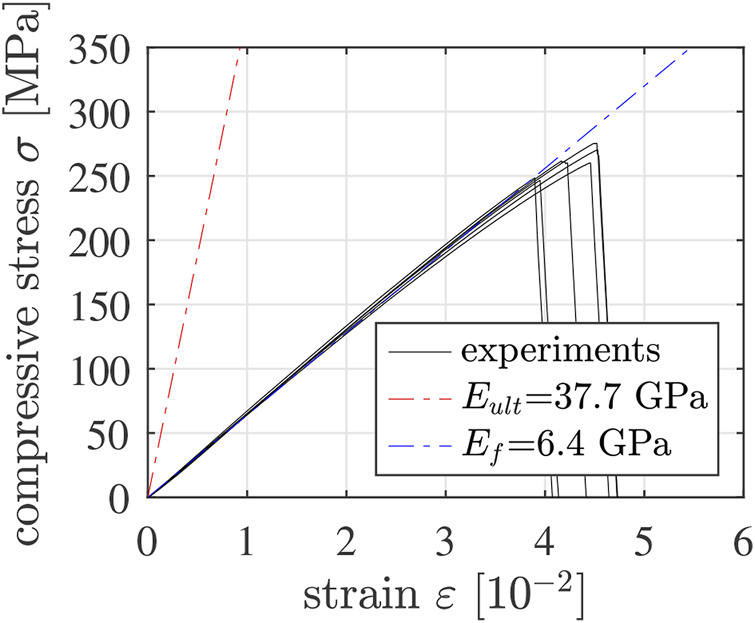
Stress-strain diagrams of the most mature 5/10 specimens: the pre-peak paths have a slope amounting to 6.4 GPa, see the blue dash-dotted line; the slope of the red dash-dotted line corresponds to actual modulus of elasticity of mature Biodentine, obtained from ultrasonic pulse velocity measurements. Note that in case of the mechanical experiments, “strain” refers to the sum of the absolute values of the compressive axial strain undergone by the specimen and the tensile axial strain undergone by the testing machine.

The actual modulus of elasticity of mature Biodentine amounts to 37.7 GPa. This value was as quantified by means of ultrasonic pulse velocity measurements, see (Dohnalík et al., 2022). The initial slope of the stress-strain graphs of the strength tests of the most mature specimens amounts to 6.4 GPa only, see Figure 14. The described difference underlines that the “machine displacements” Δ*L*(*t*) indeed account not only for the deformation of the tested specimens, but also for the deformation of the loaded parts of the testing machine.

### 5.3 Similarities and differences between construction “cement pastes” and Biodentine

Grid nanoindentation testing of Biodentine and of chemically comparable construction “cement pastes,” respectively, have shown that both types of materials consist of two populations of hydrates (= products of the chemical reaction between calcium-silicates and water). As for construction “cement pastes,” low-density calcium-silicate hydrates (C-S-H) are distinguished from high-density C-S-H (Jennings, 2000; Tennis and Jennings, 2000; Constantinides and Ulm, 2004). Biodentine, in turn, consists of LDCR hydrates and HDCR hydrates (Dohnalík et al., 2021). The microstructure of construction “cement pastes” typically consists of two times more low-density than high-density C-S-H (Constantinides and Ulm, 2004), and the strength properties of low-density C-S-H (Sarris and Constantinides, 2013) can be upscaled to explain the macroscopic strength of construction “cement pastes” (Pichler and Hellmich, 2011; Pichler et al., 2013b) and of related concretes (Königsberger et al., 2018). The microstructure of Biodentine, in turn, consists of six times more HDCR than LDCR hydrates (Dohnalík et al., 2021), and the macroscopic strength of Biodentine is significantly larger than that of construction “cement pastes”.

The described similarities and differences suggest that the superior macrostrength of Biodentine is triggered by microscopic failure of the HDCR rather than the LDCR hydrates. In addition, the sudden brittle failure of Biodentine indicates that a large portion of the HDCR hydrates fail as soon as the macroscopic loading reaches the strength of the material.

## 6 Conclusion

From the results of the presented study, the following conclusion are drawn:

• Biodentine exhibits noticable pre-peak non-linearities only during the first 90 min after production. After that, Biodentine behaves virtually linear elastically, all the way up to sudden brittle failure.
• Very young specimens showed axial splitting. At more mature material ages amounting to 1 day and more, the specimens broke into many fragments, rendering the identification of crack propagation directions impossible.
• The strength evolution of Biodentine is qualitatively similar to that of construction concretes. Quantitatively, the strength evolution of Biodentine is significantly faster and leads to a by far larger “final” strength compared to chemically comparable construction “cement pastes”.
• The smaller the length-to-diameter ratio (“slenderness ratio”) of cylindrical specimens of Biodentine, the larger is the maximum force sustained by these specimens when crushed under uniaxial compression.
• Microscopic stress states in both populations of hydrates of Biodentine, computed by means of a micromechanics model accounting for lognormal stiffness distributions of two populations of hydrates, are, in good approximation, *uniaxial* compressive stress states.
• Correlating quantiles of two lognormal distributions results in a power-law relation between the two statistical variables.
• Given that indentation modulus *and* hardness of both populations of hydrates of Biodentine are lognormally distributed, the probability density function of the effective shear strength of the high-density calcite-reinforced hydrates of Biodentine was introduced to be of lognormal nature as well.
• Assuming that the macroscopic strength of mature Biodentine results from failure of the high-density calcite-reinforced hydrates with indentation modulus equal to the median value, it was found that some 63% of the volume of Biodentine fail at virtually the same macroscopic loading. This underlines the highly optimized nature of the studied material.

## Data Availability

The raw data supporting the conclusion of this article will be made available by the authors, without undue reservation.

## Footnotes

1 In the construction sector, “cement paste” is the name not only given to freshly mixed suspension consisting of cement and water, but also to solids developing out of these suspensions.

2 So far, the sign convention used in material testing of cementitious materials was used: compression was quantified by positive numbers. From now on, the sign convention used in continuum mechanics is used: compressive normal stresses are quantified by negative numbers, tensile normal stresses are positive.

## Quantile-based correlation between two lognormal distributions

Solving both Eqs 35, 36 for *u*_*p*_ and setting the two resulting expressions equal to each other yields

$$\frac{1}{\sigma_{X}}\left[\ln\left(X_{p}\right)-\mu_{X}\right]=\frac{1}{\sigma_{Y}}\left[\ln\left(Y_{p}\right)-\mu_{Y}\right].$$
(A1)

Solving the Eq. A1 for ln(*Y*_*p*_) yields

$$\ln\left(Y_{p}\right)=\mu_{Y}+\frac{\sigma_{Y}}{\sigma_{X}}\left[\ln\left(X_{p}\right)-\mu_{X}\right].$$
(A2)

Re-arranging terms and making use of “*a* ln(*b*) = ln(*b* ^*a*^)” on the right side of Eq. A2 yields

$$\ln\left(Y_{p}\right)=\left(\mu_{Y}-\frac{\sigma_{Y}}{\sigma_{X}}\;\mu_{X}\right)+\ln\left(X_{p}^{\frac{\sigma_{Y}}{\sigma_{X}}}\right).$$
(A3)

Applying to the term in the first pair of brackets on the right side of Eq. A3, first the exponential function, and then its inverse, the natural logarithm, yields

$$\ln\left(Y_{p}\right)=\ln\left(\exp\left(\mu_{Y}-\frac{\sigma_{Y}}{\sigma_{X}}\;\mu_{X}\right)\right)+\ln\left(X_{p}^{\frac{\sigma_{Y}}{\sigma_{X}}}\right).$$
(A4)

Making use of “ln(*a*) + ln(*b*) = ln(*a* × *b*)” on the right side of Eq. A4 yields

$$\ln\left(Y_{p}\right)=\ln\left(\exp\left(\mu_{Y}-\frac{\sigma_{Y}}{\sigma_{X}}\;\mu_{X}\right)\times X_{p}^{\frac{\sigma_{Y}}{\sigma_{X}}}\right).$$
(A5)

Applying the exponential function to both sides of Eq. A5 finally yields

$$Y_{p}=\exp\left(\mu_{Y}-\frac{\sigma_{Y}}{\sigma_{X}}\;\mu_{X}\right)\times X_{p}^{\frac{\sigma_{Y}}{\sigma_{X}}}.$$
(A6)

Given that Eq. A6 is valid for any *p*-quantile, the indices *p* can be omitted. This leads to Eq. 37.

## References

[B1] American Society for Testing and Materials (2021). Standard test method for compressive strength of cylindrical concrete specimens, ASTM standard C39/C39m–21.

[B2] Amieur M. (1994). Etude numérique et expérimentale des effets d’échelle et de conditions aux limites sur des éprouvettes de béton n’ayant pas le volume représentatif [Numerical and experimental study of scale effects and boundary conditions on concrete specimens having no representative volume]. École Polytechnique Fédérale de Lausanne EPFL. Ph.D. thesis.

[B3] Atmeh A. R. (2020). Investigating the effect of bicarbonate ion on the structure and strength of calcium silicate-based dental restorative material—biodentine. Clin. Oral Investig. 24, 4597–4606. 10.1007/s00784-020-03328-y 32458073

[B4] Ausweger M. Binder E. Lahayne O. Reihsner R. Maier G. Peyerl M. (2019). Early-age evolution of strength, stiffness, and non-aging creep of concretes: Experimental characterization and correlation analysis. Materials 12, 207. 10.3390/ma12020207 30634498 PMC6357154

[B5] Barré Saint-Venant A. (1855). Mémoire sur la torsion des prismes, [Essay on twisting prisms], Mémoires des savants étrangers [Essays of foreign scholars]. Comptes Rendus l’Académie Sci. 14, 233–560.

[B7] Bernard O. Ulm F.-J. Lemarchand E. (2003). A multiscale micromechanics-hydration model for the early-age elastic properties of cement-based materials. Cem. Concr. Res. 33, 1293–1309. 10.1016/s0008-8846(03)00039-5

[B8] Budiansky B. O’Connell R. J. (1976). Elastic moduli of a cracked solid. Int. J. Solids Struct. 12, 81–97. 10.1016/0020-7683(76)90044-5

[B9] Butt N. Talwar S. Chaudhry S. Nawal R. R. Yadav S. Bali A. (2014). Comparison of physical and mechanical properties of mineral trioxide aggregate and Biodentine. Indian J. Dent. Res. 25, 692. 10.4103/0970-9290.152163 25728098

[B10] Camilleri J. Sorrentino F. Damidot D. (2013). Investigation of the hydration and bioactivity of radiopacified tricalcium silicate cement, Biodentine and MTA Angelus. Dent. Mater. 29, 580–593. 10.1016/j.dental.2013.03.007 23537569

[B11] Carde C. Francois R. (1999). Modelling the loss of strength and porosity increase due to the leaching of cement pastes. Cem. Concr. Compos. 21, 181–188. 10.1016/s0958-9465(98)00046-8

[B12] Constantinides G. Chandran K. R. Ulm F.-J. Van Vliet K. (2006). Grid indentation analysis of composite microstructure and mechanics: Principles and validation. Mater. Sci. Eng. A 430, 189–202. 10.1016/j.msea.2006.05.125

[B13] Constantinides G. Ulm F.-J. (2004). The effect of two types of C-S-H on the elasticity of cement-based materials: Results from nanoindentation and micromechanical modeling. Cem. Concr. Res. 34, 67–80. 10.1016/s0008-8846(03)00230-8

[B14] Dawood A. Manton D. Parashos P. Wong R. Palamara J. Stanton D. (2015). The physical properties and ion release of CPP-ACP-modified calcium silicate-based cements. Aust. Dent. J. 60, 434–444. 10.1111/adj.12255 25424362

[B15] Deudé V. Dormieux L. Kondo D. Maghous S. (2002). Micromechanical approach to nonlinear poroelasticity: Application to cracked rocks. J. Eng. Mech. 128, 848–855. 10.1061/(asce)0733-9399(2002)128:8(848)

[B16] Diamond S. Bonen D. (1993). Microstructure of hardened cement paste—A new interpretation. J. Am. Ceram. Soc. 76, 2993–2999. 10.1111/j.1151-2916.1993.tb06600.x

[B17] Dohnalík P. Hellmich C. Richard G. Pichler B. L. A. (2022). Stiffness and stress fluctuations in dental cement paste: A continuum micromechanics approach. Mech. Adv. Mater. Struct., 1–19. 10.1080/15376494.2022.2073493

[B18] Dohnalík P. Pichler B. L. A. Zelaya-Lainez L. Lahayne O. Richard G. Hellmich C. (2021). Micromechanics of dental cement paste. J. Mech. Behav. Biomed. Mater. 124, 104863. 10.1016/j.jmbbm.2021.104863 34634693

[B19] Donnelly E. Baker S. P. Boskey A. L. van der Meulen M. C. (2006). Effects of surface roughness and maximum load on the mechanical properties of cancellous bone measured by nanoindentation. J. Biomed. Mater. Res. Part A 77, 426–435. 10.1002/jbm.a.30633 PMC1502375 16392128

[B20] Dormieux L. Kondo D. Ulm F.-J. (2006). Microporomechanics. John Wiley & Sons. ISBN: 978-0-4700-3199-5.

[B22] Elnaghy A. M. (2014). Influence of acidic environment on properties of biodentine and white mineral trioxide aggregate: A comparative study. J. Endod. 40, 953–957. 10.1016/j.joen.2013.11.007 24935542

[B24] Fischer I. Pichler B. Lach E. Terner C. Barraud E. Britz F. (2014). Compressive strength of cement paste as a function of loading rate: Experiments and engineering mechanics analysis. Cem. Concr. Res. 58, 186–200. 10.1016/j.cemconres.2014.01.013

[B25] Grazziotin-Soares R. Nekoofar M. H. Davies T. Hübler R. Meraji N. Dummer P. M. (2019). Crystalline phases involved in the hydration of calcium silicate-based cements: Semi-quantitative Rietveld X-ray diffraction analysis. Aust. Endod. J. 45, 26–32. 10.1111/aej.12226 28857353

[B27] Hussey R. J. Wilson J. (1998). Advanced technical ceramics directory and databook. Springer Science & Business Media. ISBN: 978-1-4419-8662-7.

[B28] International Federation for Structural Concrete (2010). Fib model Code for concrete structures 2010. Ernst & Sohn: Berlin, Germany.

[B29] Irfan-ul-Hassan M. Pichler B. Reihsner R. Hellmich C. (2016). Elastic and creep properties of young cement paste, as determined from hourly repeated minute-long quasi-static tests. Cem. Concr. Res. 82, 36–49. 10.1016/j.cemconres.2015.11.007

[B30] Jang Y.-E. Lee B.-N. Koh J.-T. Park Y.-J. Joo N.-E. Chang H.-S. (2014). Cytotoxicity and physical properties of tricalcium silicate-based endodontic materials. Restor. Dent. Endod. 39, 89–94. 10.5395/rde.2014.39.2.89 24790920 PMC3978109

[B31] Jennings H. M. (2000). A model for the microstructure of calcium silicate hydrate in cement paste. Cem. Concr. Res. 30, 101–116. 10.1016/s0008-8846(99)00209-4

[B32] Karte P. Hlobil M. Reihsner R. Dörner W. Lahayne O. Eberhardsteiner J. (2015). Unloading-based stiffness characterisation of cement pastes during the second, third and fourth day after production. Strain 51, 156–169. 10.1111/str.12129

[B33] Kayahan M. B. Nekoofar M. H. McCann A. Sunay H. Kaptan R. F. Meraji N. (2013). Effect of acid etching procedures on the compressive strength of 4 calcium silicate–based endodontic cements. J. Endod. 39, 1646–1648. 10.1016/j.joen.2013.09.008 24238465

[B34] Keskin C. Sariyilmaz E. KeleŞ A. (2019). The effect of bleaching agents on the compressive strength of calcium silicate-based materials. Aust. Endod. J. 45, 311–316. 10.1111/aej.12318 30338618

[B36] Königsberger M. Hellmich C. Pichler B. (2016). Densification of C-S-H is mainly driven by available precipitation space, as quantified through an analytical cement hydration model based on NMR data. Cem. Concr. Res. 88, 170–183. 10.1016/j.cemconres.2016.04.006

[B37] Königsberger M. Hlobil M. Delsaute B. Staquet S. Hellmich C. Pichler B. (2018). Hydrate failure in itz governs concrete strength: A micro-to-macro validated engineering mechanics model. Cem. Concr. Res. 103, 77–94. 10.1016/j.cemconres.2017.10.002

[B38] Königsberger M. Zelaya-Lainez L. Lahayne O. Pichler B. L. Hellmich C. (2021). Nanoindentation-probed Oliver-Pharr half-spaces in alkali-activated slag-fly ash pastes: Multimethod identification of microelasticity and hardness. Mech. Adv. Mater. Struct. 29, 4878–4889. 10.1080/15376494.2021.1941450

[B40] Li Q. Hurt A. P. Coleman N. J. (2019). The application of 29Si NMR spectroscopy to the analysis of calcium silicate-based cement using Biodentine™ as an Example^29^Si NMR spectroscopy to the analysis of calcium silicate-based cement using biodentine as an example. J. Funct. Biomaterials 10, 25. 10.3390/jfb10020025 PMC6617092 31151191

[B41] Ma Y. Ye G. Hu J. (2017). Micro-mechanical properties of alkali-activated fly ash evaluated by nanoindentation. Constr. Build. Mater. 147, 407–416. 10.1016/j.conbuildmat.2017.04.176

[B42] Miller M. Bobko C. Vandamme M. Ulm F.-J. (2008). Surface roughness criteria for cement paste nanoindentation. Cem. Concr. Res. 38, 467–476. 10.1016/j.cemconres.2007.11.014

[B43] Moreno-Vargas Y. A. Luna-Arias J. P. Flores-Flores J. O. Orozco E. Bucio L. (2017). Hydration reactions and physicochemical properties in a novel tricalcium-dicalcium silicate-based cement containing hydroxyapatite nanoparticles and calcite: A comparative study. Ceram. Int. 43, 13290–13298. 10.1016/j.ceramint.2017.07.027

[B45] Natale L. Rodrigues M. Xavier T. Simões A. De Souza D. Braga R. (2015). Ion release and mechanical properties of calcium silicate and calcium hydroxide materials used for pulp capping. Int. Endod. J. 48, 89–94. 10.1111/iej.12281 24646329

[B46] Oliver W. C. Pharr G. M. (1992). An improved technique for determining hardness and elastic modulus using load and displacement sensing indentation experiments. J. Mater. Res. 7, 1564–1583. 10.1557/jmr.1992.1564

[B47] Pensée V. Kondo D. Dormieux L. (2002). Micromechanical analysis of anisotropic damage in brittle materials. J. Eng. Mech. 128, 889–897. 10.1061/(asce)0733-9399(2002)128:8(889)

[B48] Pichler B. Hellmich C. (2011). Upscaling quasi-brittle strength of cement paste and mortar: A multi-scale engineering mechanics model. Cem. Concr. Res. 41, 467–476. 10.1016/j.cemconres.2011.01.010

[B49] Pichler B. Hellmich C. Eberhardsteiner J. (2009). Spherical and acicular representation of hydrates in a micromechanical model for cement paste: Prediction of early-age elasticity and strength. Acta Mech. 203, 137–162. 10.1007/s00707-008-0007-9

[B50] Pichler B. Hellmich C. Eberhardsteiner J. Wasserbauer J. Termkhajornkit P. Barbarulo R. (2013a). Effect of gel–space ratio and microstructure on strength of hydrating cementitious materials: An engineering micromechanics approach. Cem. Concr. Res. 45, 55–68. 10.1016/j.cemconres.2012.10.019

[B51] Pichler B. Hellmich C. Eberhardsteiner J. Wasserbauer J. Termkhajornkit P. Barbarulo R. (2013b). “The counteracting effects of capillary porosity and of unhydrated clinker grains on the macroscopic strength of hydrating cement paste–a multiscale model,” in 9th International Conference on Creep, Shrinkage, and Durability Mechanics (CONCREEP-9), 40–47. 10.1061/9780784413111.004

[B52] Pires M. D. Cordeiro J. Vasconcelos I. Alves M. Quaresma S. A. Ginjeira A. (2021). Effect of different manipulations on the physical, chemical and microstructural characteristics of Biodentine. Dent. Mater. 37, e399–e406. 10.1016/j.dental.2021.03.021 33863567

[B53] Primus C. M. Tay F. R. Niu L. (2019). Bioactive tri/dicalcium silicate cements for treatment of pulpal and periapical tissues. Acta Biomater. 96, 35–54. 10.1016/j.actbio.2019.05.050 31146033 PMC6717675

[B54] Rajasekharan S. Martens L. Cauwels R. Anthonappa R. P. (2018). Biodentine^TM^ material characteristics and clinical applications: A 3 year literature review and update. Eur. Archives Paediatr. Dent. 19, 1–22. 10.1007/s40368-018-0328-x 29372451

[B55] Rajasekharan S. Martens L. Cauwels R. Verbeeck R. (2014). Biodentine^TM^ material characteristics and clinical applications: A review of the literature. Eur. Archives Paediatr. Dent. 15, 147–158. 10.1007/s40368-014-0114-3 24615290

[B56] Sarris E. Constantinides G. (2013). Finite element modeling of nanoindentation on C–S–H: Effect of pile-up and contact friction. Cem. Concr. Compos. 36, 78–84. 10.1016/j.cemconcomp.2012.10.010

[B57] Septodont (2014). BiodentineTM Dentinersatz für die Zahnerhaltung (Septodont GmbH). ISBN: 978-3-00-046703-5.

[B58] Shiotani T. Bisschop J. Van Mier J. (2003). Temporal and spatial development of drying shrinkage cracking in cement-based materials. Eng. Fract. Mech. 70, 1509–1525. 10.1016/s0013-7944(02)00150-9

[B59] Subramanyam D. Vasantharajan M. (2017). Effect of oral tissue fluids on compressive strength of MTA and biodentine: An *in vitro* study. J. Clin. Diagnostic Res. JCDR 11, ZC94–ZC96. 10.7860/jcdr/2017/24510.9722 PMC5449928 28571272

[B60] Taplin J. (1959). A method for following the hydration reaction in Portland cement paste. Aust. J. Appl. Sci. 10, 329–345.

[B61] Tennis P. D. Jennings H. M. (2000). A model for two types of calcium silicate hydrate in the microstructure of Portland cement pastes. Cem. Concr. Res. 30, 855–863. 10.1016/s0008-8846(00)00257-x

[B62] Zaoui A. (2002). Continuum micromechanics: Survey. J. Eng. Mech. 128, 808–816. 10.1061/(asce)0733-9399(2002)128:8(808)

[B63] Zhu Q.-Z. Shao J. Kondo D. (2011). A micromechanics-based thermodynamic formulation of isotropic damage with unilateral and friction effects. Eur. J. Mech. - A/Solids 30, 316–325. 10.1016/j.euromechsol.2010.12.005

