# Supplementary material for: Strength of a cement-based dental material: Early age testing and first micromechanical modeling at mature age
Source: Front Bioeng Biotechnol. 2023 Mar 9;11:1047470. doi: 10.3389/fbioe.2023.1047470 (PMC10044622; doi:10.3389/fbioe.2023.1047470)
Supplement: Supplementary file 1 [file Presentation1.pdf]

# *Supplementary Material*

## 1 DIMENSIONS OF TESTED SPECIMENS

**Table S1.** Diameters and lengths of the 5/10 specimens measured after polishing of the contact surfaces; every listed value is the arithmetic mean of three measurements.

| mat. | diameter $D$ [mm] of a specimen $n$ | | | | | | | length $L$ [mm] of a specimen $n$ | | | | | | |
|---|---|---|---|---|---|---|---|---|---|---|---|---|---|---|
| age | 1 | 2 | 3 | 4 | 5 | 6 | 7 | 1 | 2 | 3 | 4 | 5 | 6 | 7 |
| 1 hr. | 5.01 | 5.00 | 5.00 | 5.00 | 4.98 | 4.96 | – | 9.37 | 9.25 | 9.42 | 9.20 | 9.13 | 8.88 | – |
| 1.5 hrs. | 5.01 | 5.00 | 5.01 | 5.00 | 5.02 | 5.00 | 4.99 | 9.30 | 9.33 | 9.33 | 9.46 | 9.37 | 9.30 | 9.35 |
| 3 hrs. | 4.98 | 5.00 | 4.99 | 4.99 | 4.99 | 4.99 | – | 9.07 | 9.36 | 9.44 | 9.07 | 9.27 | 9.14 | – |
| 7.5 hrs. | 4.99 | 5.00 | 5.01 | 5.02 | 5.00 | 4.98 | 4.98 | 9.47 | 8.54 | 9.32 | 9.25 | 9.31 | 8.98 | 8.74 |
| 25.5 hrs. | 5.01 | 4.98 | 4.99 | 4.99 | 5.02 | 5.00 | 5.04 | 9.09 | 9.45 | 9.02 | 9.37 | 8.99 | 9.20 | 9.04 |
| 7 days | 4.99 | 4.99 | 4.99 | 4.98 | 4.95 | 5.00 | 5.01 | 8.96 | 9.25 | 9.24 | 9.50 | 8.84 | 9.13 | 9.29 |
| 7 days* | 4.98 | 4.98 | 5.01 | 5.00 | 5.00 | 4.98 | 5.02 | 9.21 | 9.29 | 9.07 | 9.28 | 9.36 | 9.32 | 9.26 |
| 14 days | 4.97 | 5.01 | 4.99 | 4.99 | 4.99 | 5.03 | 4.99 | 8.84 | 9.32 | 9.25 | 9.15 | 9.11 | 9.14 | 9.27 |
| 14 days* | 5.01 | 5.00 | 5.02 | 5.02 | 5.00 | 5.00 | 5.01 | 9.36 | 9.47 | 9.34 | 9.20 | 9.48 | 9.11 | 9.09 |
| 21 days | 5.02 | 5.01 | 5.00 | 5.03 | 5.03 | 5.00 | 5.02 | 9.38 | 9.44 | 9.33 | 9.47 | 9.20 | 9.11 | 9.10 |
| 28 days | 5.01 | 5.02 | 5.01 | 4.99 | 5.00 | 5.03 | 5.02 | 9.15 | 9.12 | 9.25 | 9.19 | 9.22 | 9.19 | 9.36 |

* the numbering of the specimens continues as $n + 7$.

**Table S2.** Diameters and lengths of the 4/6 specimens measured after polishing of the contact surfaces; every listed value is the arithmetic mean of three measurements.

| mat. | diameter $d$ [mm] of a specimen $n$ | | | | | | | length $L$ [mm] of a specimen $n$ | | | | | | |
|---|---|---|---|---|---|---|---|---|---|---|---|---|---|---|
| age | 1 | 2 | 3 | 4 | 5 | 6 | 7 | 1 | 2 | 3 | 4 | 5 | 6 | 7 |
| 1.5 hrs. | 3.89 | 3.98 | 3.99 | 3.98 | 3.91 | 3.93 | 3.91 | 5.04 | 4.97 | 5.32 | 5.48 | 5.43 | 5.41 | 4.99 |
| 7.5 hrs. | –‡ | 3.88 | 3.86 | 3.95 | 3.99 | 3.94 | 3.98 | –‡ | 5.17 | 5.69 | 5.31 | 5.38 | 5.22 | 5.43 |
| 25.5 hrs. | 3.94 | 3.96 | 3.89 | 3.90 | 3.94 | 3.90 | 3.90 | 5.63 | 5.27 | 5.46 | 5.45 | 5.41 | 5.44 | 5.68 |
| 7 days | 3.91 | 3.92 | 3.98 | 3.96 | 3.90 | 3.94 | 3.91 | 5.27 | 5.51 | 5.34 | 5.45 | 5.25 | 5.51 | 5.13 |
| 14 days | 3.91 | 3.88 | 3.93 | 3.89 | 4.04 | 4.00 | 3.93 | 5.47 | 4.30 | 5.09 | 5.18 | 5.36 | 5.01 | 4.73 |
| 21 days | 3.88 | 3.95 | 3.96 | 3.99 | 3.91 | –‡ | 4.01 | 5.25 | 5.12 | 5.24 | 4.60 | 5.26 | –‡ | 5.49 |
| 28 days | 3.89 | 3.96 | 3.97 | 3.91 | 3.93 | 3.89 | 3.99 | 5.17 | 5.21 | 5.49 | 5.17 | 5.10 | 5.14 | 5.17 |

‡ specimens failed during handling, prior to testing

## 2 ANALYSIS OF THE EQUIVALENT SHEAR STRENGTH OF THE LDCR HYDRATES

In the following, it is checked whether or not the LDCR hydrates could be the microstructural origin of macroscopic failure of mature Biodentine subjected to uniaxial compression. To this end, the macroscopic uniaxial compressive strength, see Eq. (30), is downscaled according to Eqs. (21)–(24) into the LDCR hydrates, see Fig. S1.

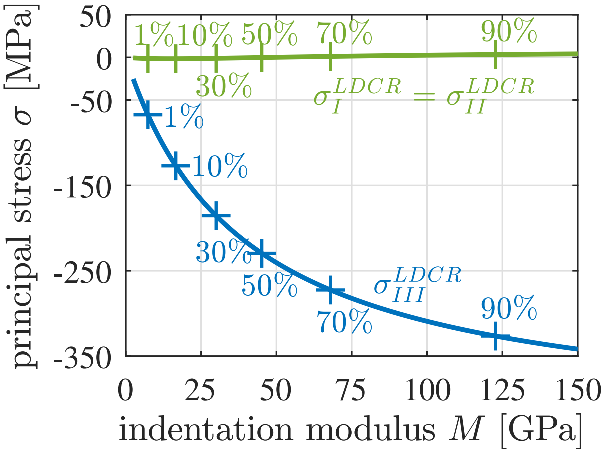


**Fig. S1.** Axial and lateral components of the microscopic principal stress states experienced by less-dense calcite-reinforced hydrates, as a function of the indentation modulus $M$.

The correlation between the lognormal distributions of the indentation modulus $M$ and the indentation hardness $H$ of the LDCR hydrates is illustrated in Fig. S2(a), (b). In the same way, the known lognormal distribution of the indentation modulus $M$ is correlated with the sought lognormal distribution of the equivalent shear strength $C$ of the LDCR hydrates, see Eq. (38). Realistic values of $\mu_C$ and $\sigma_C$ are identified such that the corresponding straight line in Fig. S2(c) becomes a tangent to the graph showing $|\sigma_{axi}/2|$ over $M$. The contact point refers to the $50\%$ quantile of $M$. As for the LDCR hydrates, this value amounts to $45.1\,\mathrm{GPa}$, see Fig. S2(c) and Table 7. The corresponding lognormal parameters of the distribution of the equivalent shear strength read as

$$\mu_C = 4.737\,, \tag{S1}$$
$$\sigma_C = 0.365\,. \tag{S2}$$

The corresponding probability density function of the equivalent shear strength of the LDCR hydrates is illustrated in Fig. S2(d). Related values of the mode, the median, and the mean value are listed in Table S3.

**Table S3.** Mode, median, and mean value of the lognormal distribution of the equivalent shear strength $C$ of the LDCR hydrates, see also Eqs. (S1) and (S2).

| mode [MPa] | median [MPa] | mean [MPa] |
|---|---|---|
| 99.9 | 114.1 | 122.0 |

The degree of utilization of all LDCR hydrates is larger than or equal to some $78\%$. This is smaller than the utilization degree of the HDCR hydrates, compare Figs. S3(a) and 13(a). All LDCR hydrates between

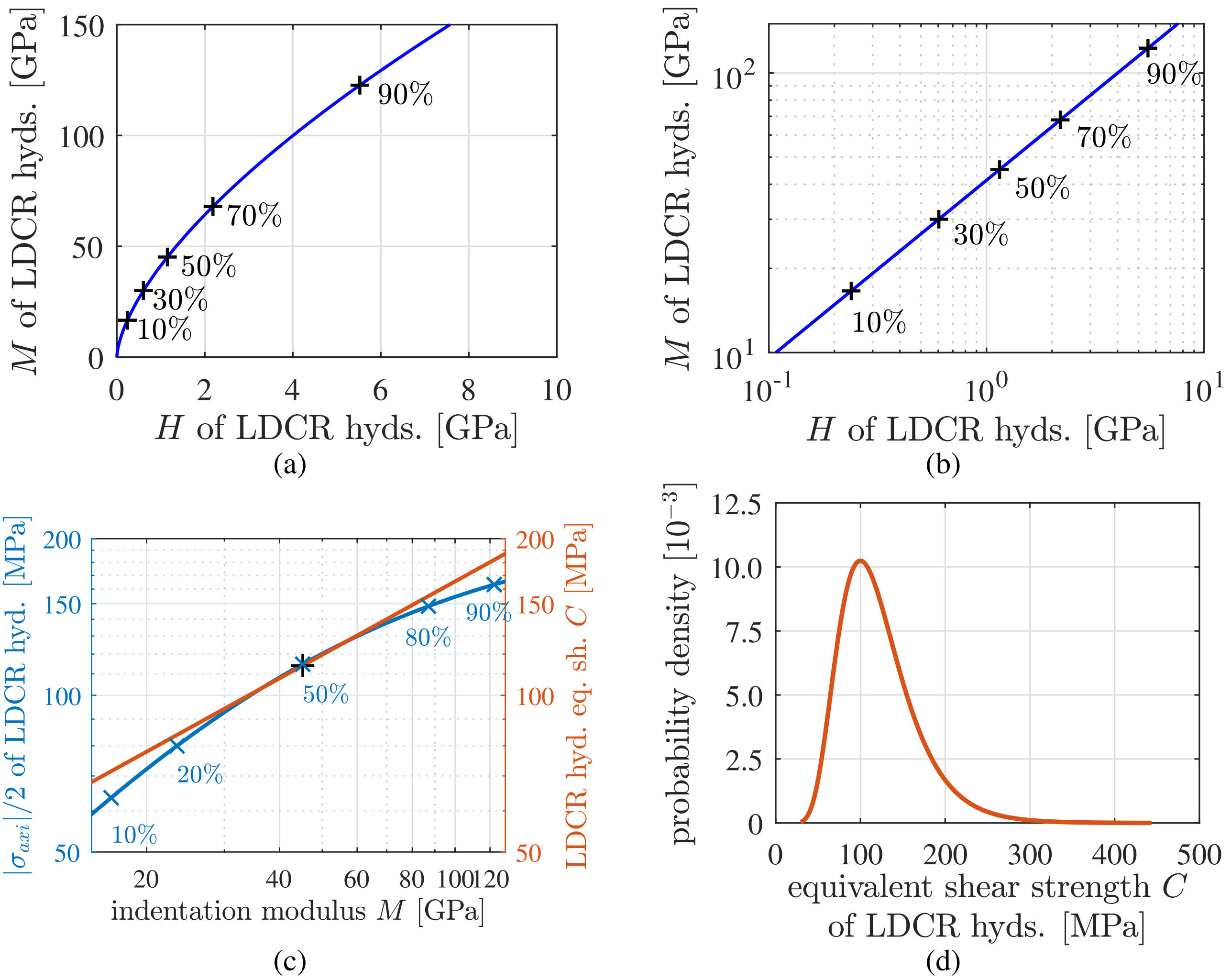


**Fig. S2.** (a), (b) Quantile-based power-law correlation between the lognormal distributions of the indentation modulus (see the ordinates) and the indentation hardness (see the abscissas) of the LDCR hydrates: representation of the power-law-relation according to Eq. (37) with $X = H$, $Y = M$, $\mu_M = 3.81$, $\sigma_M = 0.78$, $\mu_H = 0.14$, and $\sigma_H = 1.23$, see Table 8 and Dohnalík et al. (2021): (a) natural scale, as well as (b) double-logarithmic representation; the black "+" symbols mark $p$-quantiles, with values of $p$ equal to the printed percentage values. (c) Illustration of the failure criterion (34): the equivalent shear strength of all LDCR hydrates is larger than $|\sigma_{axi}/2|$, except for the median value of the indentation modulus, for which $|\sigma_{axi}/2| = C$; the blue "+" symbols mark $p$-quantiles, with values of $p$ given as percentage values. (d) Lognormal probability density function of the equivalent shear strength of the LDCR hydrates; lognormal parameters of the distribution are given in Eqs. (S1) and (S2).

the 36.1%-quantile and the 64.6%-quantile have degrees of utilization larger than or equal to 99%, see Fig. S3(b). Therefore, if the lognormal distribution of the equivalent shear strength according to Fig. S2(d) is realistic, then $64.6\% - 36.1\% = 28.5\%$ of the LDCR hydrates have a degree of utilization larger than or equal to 99% and will, therefore, fail virtually simultaneously. Given that LDCR hydrates make up 12.3% of the volume of Biodentine, see Table 8, $28.5\% \times 12.3\% = 3.5\%$ of the volume of Biodentine will fail at the same time. This does not propose the LDCR hydrates as primary reason for the sudden, well-spread brittle failure of the mature Biodentine specimens under destructive compressive mechanical testing.

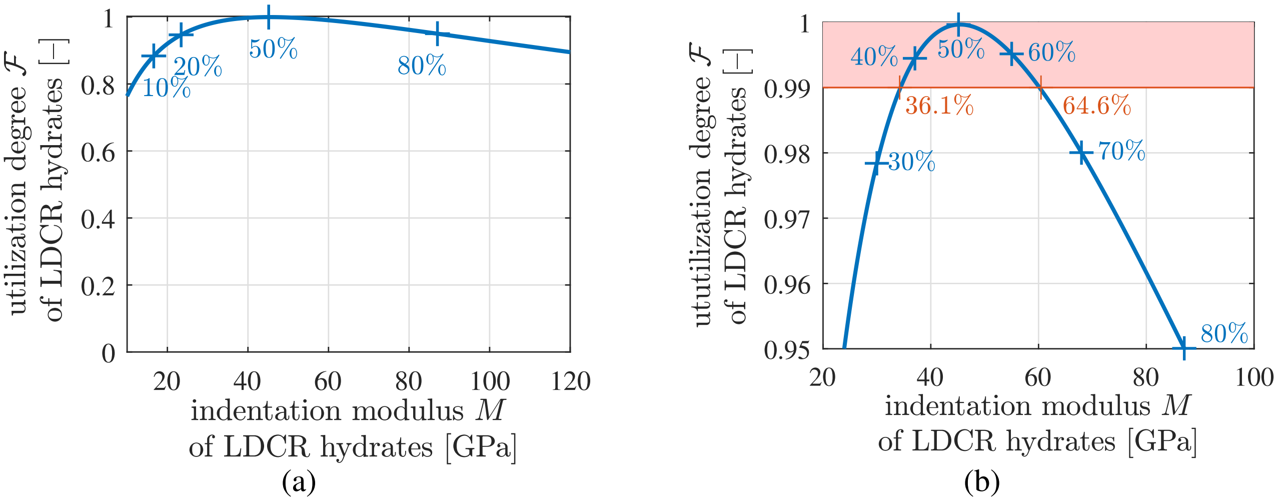


**Fig. S3.** Degree of utilization $\mathcal{F} = |\sigma_{axi}|/(2C)$ as a function of the indentation modulus $M$ of LDCR hydrates; the "+" symbols mark $p$-quantiles, with values of $p$ given as percentage values.

## 3 FUNDAMENTALS OF STIFFNESS HOMOGENIZATION

Methods of continuum micromechanics allow for stiffness homogenization of representative volume elements (RVE) of microheterogeneous materials. The RVEs fulfil the scale separation principle, i.e. the characteristic size of the microheterogeneities is at least 2 to 3 times smaller than the characteristic size of the RVE (Drugan and Willis, 1996), and the characteristic size of the RVE is by a factor of 5 to 10 smaller than the characteristic size of the structure containing the RVE and/or the characteristic length of the external loading imposed on that structure (Kohlhauser and Hellmich, 2013). Inside the RVE, field equations of linear elasticity are considered. At the boundary of the RVE, uniform strain boundary conditions are imposed

$$\underline{u}(\underline{x}) = \mathbf{E} \cdot \underline{x}\,, \tag{S3}$$

where $\underline{u}$ denotes the displacement vector, $\underline{x}$ the position vector, and $\mathbf{E}$ the imposed uniform macrostrain state.

The heterogeneous microstructure of composites is frequently so gracefully built that it cannot be represented in full detail. As a remedy, the volume of the studied RVE, $V_{RVE}$, is subdivided into (not necessarily connected) subvolumes occupied by the different microstructural constituents called "material phases":

$$V_{RVE} = \sum_{i=1}^{n_p} V_i\,, \tag{S4}$$

where $n_p$ denotes the number of material phases and $V_i$ stands for the volume occupied by the $i^{\text{th}}$ material phase, such that $f_i = V_i/V_{RVE}$ quantifies its volume fraction. The material phases are selected such that the matter filling every phase volume $V_i$ is characterized by a uniform elastic stiffness tensor $\mathbb{C}_i$.

Scale transitions are made possible by means of so-called phase strain concentration tensors $\mathbb{A}_i$. They allow for (i) the macro-to-micro scale transition regarding strains (Hill, 1963), also referred to as strain-concentration and strain-downscaling:

$$\boldsymbol{\varepsilon}_i = \mathbb{A}_i : \mathbf{E}\,, \qquad i = 1, 2, \ldots, n_p\,, \tag{S5}$$

where $\varepsilon_i$ stands for the volume-averaged strain of material phase $i$, and : stands for a double-contracting tensor product, and (ii) the micro-to-macro scale transition regarding stiffness Hill (1963), also referred to as stiffness-homogenization and stiffness-upscaling

$$\mathbb{C}_{hom} = \sum_{i=1}^{n_p} f_i \, \mathbb{C}_i : \mathbb{A}_i \,, \tag{S6}$$

where $\mathbb{C}_{hom}$ denotes the homogenized elastic stiffness of the microheterogeneous material. However, because the very details of the microstructure of an RVE are unknown, phase strain concentration tensors cannot be computed up to analytical precision.

In continuum micromechanics, phase strain concentration tensors are estimated with the help of auxiliary three-dimensional matrix-inclusion problems (Eshelby, 1957; Laws, 1977). $n_p$ such problems are introduced; one for every material phase. The elastic stiffness, the ellipsoidal shape, and the orientation in space of the $i^{\text{th}}$ material phase are assigned to the inclusion of the $i^{\text{th}}$ matrix-inclusion problem, see Fig. S4. The stiffness of the infinite matrices of all these problems, $\mathbb{C}_\infty$, is equal to a characteristic stiffness

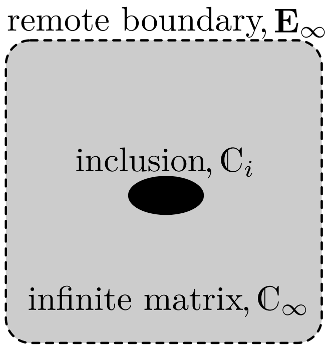


**Fig. S4.** Auxiliary matrix-inclusion problem: a three-dimensional ellipsoidal inclusion of stiffness $\mathbb{C}_i$ is embedded in an infinite matrix with stiffness $\mathbb{C}_\infty$; the matrix is subjected to uniform far-field ("remote") strains $\mathbf{E}_\infty$.

of the microheterogeneous material of interest.

- If the material of interest is a matrix-inclusion composite, the stiffness of the infinite matrices of all auxiliary matrix-inclusion problems is equal to the stiffness of the matrix of the composite of interest. This leads to so-called Mori-Tanaka schemes (Mori and Tanaka, 1973; Benveniste, 1987).
- If the material of interest is a composite with a highly disordered (= "polycristalline") arrangement of the material phases, the stiffness of the infinite matrices of all auxiliary matrix-inclusion problems is equal to the homogenized stiffness of the composite of interest. This leads to so-called self-consistent schemes (Zaoui, 2002; Bernard et al., 2003; Dormieux et al., 2006).

The infinite matrices of all auxiliary matrix-inclusion problems are subjected to the same remote strain state $\mathbf{E}_\infty$. The latter is linked to the strain $\mathbf{E}$ imposed on the RVE of the composite of interest, as explained next. The strains inside the inclusions of all matrix-inclusion problems are uniform (= spatially constant) and can be computed analytically

$$\varepsilon_i = \left[\mathbb{I} + \mathbb{P}_i : (\mathbb{C}_i - \mathbb{C}_\infty)\right]^{-1} : \mathbf{E}_\infty \,, \qquad i = 1, 2, \ldots, n_p \,, \tag{S7}$$

where $\varepsilon_i$ is the strain inside the $i^{\text{th}}$ inclusion, $\mathbb{I}$ the symmetric fourth-order identity tensor, and $\mathbb{P}_i$ the Hill tensor of the $i^{\text{th}}$ inclusion, see also Dormieux et al. (2006). It is assumed that the uniform strain in the $i^{\text{th}}$ inclusion is an estimate of the volume-averaged strain of the $i^{\text{th}}$ material phase of the composite of interest. In other words, $\varepsilon_i$ in Eq. (S7) is used as an estimate for $\varepsilon_i$ in Eq. (S5). The volume-averaged strains of the material phases of the composite of interest, in turn, must fulfill the strain average rule

$$\mathbf{E} = \sum_{i=1}^{n_p} f_i \, \varepsilon_i \,. \tag{S8}$$

Inserting $\varepsilon_i$ according to Eq. (S7) into Eq. (S8) and solving the resulting expression for $\mathbf{E}_\infty$ yields the following expression for $\mathbf{E}_\infty$ as a function of $\mathbf{E}$

$$\mathbf{E}_\infty = \left[ \sum_{j=1}^{n_p} f_j \, \left[ \mathbb{I} + \mathbb{P}_j^\infty : (\mathbb{C}_j - \mathbb{C}_\infty) \right]^{-1} \right]^{-1} : \mathbf{E} \,. \tag{S9}$$

Insertion of $\mathbf{E}_\infty$ according to Eq. (S9) into Eq. (S7), and comparison of the resulting expression with Eq. (S5) yields the following estimate of the phase strain concentration tensors:

$$\mathbb{A}_i = \left[ \mathbb{I} + \mathbb{P}_i^\infty : (\mathbb{C}_i - \mathbb{C}_\infty) \right]^{-1} : \left[ \sum_{j=1}^{n_p} f_j \, \left[ \mathbb{I} + \mathbb{P}_j^\infty : (\mathbb{C}_j - \mathbb{C}_\infty) \right]^{-1} \right]^{-1} . \tag{S10}$$

Four characteristic features of heterogeneous material have to be know prior to applying Eq. (S10). These features account for elastic stiffness of the material phases, their shapes, orientations, volume fractions, and interactions among them. As regards the latter, two distinct types of interactions are distinguished: polycrystalline and matrix-inclusion type. Polycrystalline interaction, refers to perfectly disordered arrangement of material phases. This manifests in Eq. (S10) in a way that the $\mathbb{C}_\infty$ is equal to stiffness of the homogenized RVE $\mathbb{C}_{hom}$. Matrix-inclusion type interaction considers stiffness of the infinite matrix $\mathbb{C}_\infty$ in Eq. (S10) equal to stiffness of the real RVE-related matrix (Zaoui, 2002).

## REFERENCES


Benveniste, Y. (1987). A new approach to the application of Mori-Tanaka's theory in composite materials. *Mechanics of Materials* 6, 147–157. doi:10.1016/0167-6636(87)90005-6

Bernard, O., Ulm, F.-J., and Lemarchand, E. (2003). A multiscale micromechanics-hydration model for the early-age elastic properties of cement-based materials. *Cement and Concrete Research* 33, 1293–1309. doi:10.1016/S0008-8846(03)00039-5

Dohnalík, P., Pichler, B. L. A., Zelaya-Lainez, L., Lahayne, O., Richard, G., and Hellmich, C. (2021). Micromechanics of dental cement paste. *Journal of the Mechanical Behavior of Biomedical Materials* 124, 104863. doi:10.1016/j.jmbbm.2021.104863

Dormieux, L., Kondo, D., and Ulm, F.-J. (2006). *Microporomechanics* (John Wiley & Sons). ISBN: 978-0-4700-3199-5

Drugan, W. and Willis, J. (1996). A micromechanics-based nonlocal constitutive equation and estimates of representative volume element size for elastic composites. *Journal of the Mechanics and Physics of Solids* 44, 497–524. doi:10.1016/0022-5096(96)00007-5

Eshelby, J. D. (1957). The determination of the elastic field of an ellipsoidal inclusion, and related problems. *Proceedings of the Royal Society of London: Series A* 241, 376–396. doi:10.1098/rspa.1957.0133

Hill, R. (1963). Elastic properties of reinforced solids: Some theoretical principles. *Journal of the Mechanics and Physics of Solids* 11, 357–372. doi:10.1016/0022-5096(63)90036-X

Kohlhauser, C. and Hellmich, C. (2013). Ultrasonic contact pulse transmission for elastic wave velocity and stiffness determination: Influence of specimen geometry and porosity. *Engineering Structures* 47, 115–133. doi:10.1016/j.engstruct.2012.10.027

Laws, N. (1977). The determination of stress and strain concentrations at an ellipsoidal inclusion in an anisotropic material. *Journal of Elasticity* 7, 91–97. doi:10.1007/BF00041133

Mori, T. and Tanaka, K. (1973). Average stress in matrix and average elastic energy of materials with misfitting inclusions. *Acta Metallurgica* 21, 571–574. doi:10.1016/0001-6160(73)90064-3

Zaoui, A. (2002). Continuum micromechanics: Survey. *Journal of Engineering Mechanics* 128, 808–816. doi:10.1061/(ASCE)0733-9399(2002)128:8(808)
